# μ Opioid receptor: novel antagonists and structural modeling

**DOI:** 10.1038/srep21548

**Published:** 2016-02-18

**Authors:** Teresa Kaserer, Aquilino Lantero, Helmut Schmidhammer, Mariana Spetea, Daniela Schuster

**Affiliations:** 1 Computer-Aided Molecular Design Group, Department of Pharmaceutical Chemistry, Institute of Pharmacy and Center for Molecular Biosciences Innsbruck (CMBI), University of Innsbruck, Innrain 80-82, 6020 Innsbruck, Austria; 2 Opioid Research Group, Department of Pharmaceutical Chemistry, Institute of Pharmacy and Center for Molecular Biosciences Innsbruck (CMBI), University of Innsbruck, Innrain 80-82, 6020 Innsbruck, Austria

## Abstract

The μ opioid receptor (MOR) is a prominent member of the G protein-coupled receptor family and the molecular target of morphine and other opioid drugs. Despite the long tradition of MOR-targeting drugs, still little is known about the ligand-receptor interactions and structure-function relationships underlying the distinct biological effects upon receptor activation or inhibition. With the resolved crystal structure of the β-funaltrexamine-MOR complex, we aimed at the discovery of novel agonists and antagonists using virtual screening tools, i.e. docking, pharmacophore- and shape-based modeling. We suggest important molecular interactions, which active molecules share and distinguish agonists and antagonists. These results allowed for the generation of theoretically validated *in silico* workflows that were employed for prospective virtual screening. Out of 18 virtual hits evaluated in *in vitro* pharmacological assays, three displayed antagonist activity and the most active compound significantly inhibited morphine-induced antinociception. The new identified chemotypes hold promise for further development into neurochemical tools for studying the MOR or as potential therapeutic lead candidates.

The μ opioid receptor (MOR), a member of the opioid neuromodulatory system and of the large family of G protein-coupled receptors (GPCRs), is the main pharmacological target for the management of moderate to severe pain, and is of therapeutic value for the treatment of drug abuse, alcohol abuse, and gastrointestinal motility dysfunction^1^,^2^. The MORs are integral membrane proteins widely distributed throughout the central nervous system and the periphery. They are the molecular targets of a large variety of opioid drugs, of which morphinans represent one of the main classes of ligands binding to the MOR, as well as clinically useful agents^2^,^3^,^4^.

Despite the long-lasting tradition of MOR-targeting drugs, still little is known about the difference in the receptor-ligand interaction patterns of agonists and antagonists. One common hypothesis states that the size of the N-substituent in morphinans may determine their biological activity. Accordingly, larger substituents such as allyl- or cyclopropylmethyl- at the nitrogen are commonly associated with an antagonist property, whereas agonists mainly contain a methyl group^5^,^6^. However, also ligands with larger groups at the morphinan nitrogen displaying agonist activity were described^4^,^6^,^7^,^8^,^9^, thereby challenging this theory.

In 2012, the first X-ray crystal structure of the MOR from murine origin was published in complex with the irreversible morphinan antagonist β-funaltrexamine (β-FNA) (PDB-entry 4DKL^10^), aiming to provide significant details on the ligand-receptor binding patterns at the molecular level. In the reported structure^10^, β-FNA forms polar interactions with the amino acid residues Asp147 and Tyr148, and *via* a water network also with His297. In addition, it is involved in hydrophobic interactions with the residues Tyr326, Met151, Ile296, Val300, and Trp293. Finally, β-FNA is covalently attached to the MOR *via* Lys233^10^.

It is recognized that X-ray crystal structures provide valuable insights into the binding modes of ligands, and this knowledge can be exploited with computational techniques for the identification of novel bioactive molecules. Many diverse biological effects are likely to occur upon binding of a ligand to the GPCR, such as the MOR, and *in silico* methods that can successfully distinguish between agonists and antagonists may help to shed light upon the underlying molecular mechanism of action. A plethora of different strategies and software solutions is nowadays available^11^, and the current study applied pharmacophore models generated with LigandScout^12^, shape-based models created with Rapid Overlay of Chemical Structures (ROCS)^13^,^14^, and a docking workflow with GOLD^15^,^16^ for virtual screening of a commercial compound library towards discovery of novel agonists and antagonists at the MOR. Exemplified top-ranked virtual hits were selected for biological evaluation. As a result of the virtual screening campaign, we have identified novel chemotypes that display MOR activity in competition binding and functional *in vitro* and *in vivo* assays. The molecules presented herein have MOR antagonist properties and are structurally distinct scaffolds compared to the so far known MOR ligands. The identification of new scaffolds interacting with the MOR could have a major impact on the development of superior treatments of neurological and other human disorders, and the availability of pharmacological tools for the study of this receptor system.

## Results

### Molecular modeling

#### Analysis of the mode of action and docking of ligands at the MOR

**Figure 1:**
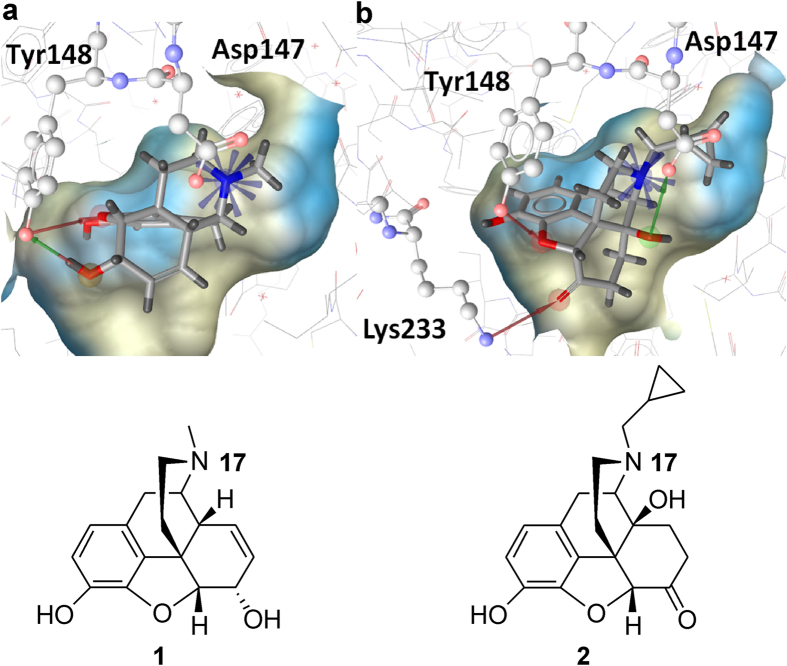
Analysis of docking poses. The docking poses of the agonist morphine **1** (**a**) and the antagonist naltrexone **2** (**b**) show common interactions with Asp147 and Tyr148. In addition, naltrexone forms a hydrogen bond with Lys233. Protein-ligand interactions are color-coded: Red arrow: hydrogen bond acceptor (HBA), green arrow: hydrogen bond donor (HBD), blue star: positively ionizable (PI). Only the relevant interactions are illustrated. The surface of the binding site is color-coded by aggregated hydrophilicity (blue)/hydrophobicity (grey). Position N17 is highlighted in the structures of the two morphinans.

One prominent hypothesis stated that the size of the substitution at the N17 position in morphinans (Fig. 1) was responsible for the different biological activity observed for MOR ligands as agonists and antagonists^5^. However, some agonists with a typical antagonist substitution pattern were also described^4^,^6^,^7^,^8^,^9^, which hampered this theory. In the absence of another hypothesis, we first aimed to generate pharmacophore- and ROCS-models based on that assumption. A pharmacophore model represents one binding mode of active compounds, and also the shape-based models generated with ROCS include electrochemical interaction features. However, the different interaction patterns of agonists and antagonists were not known, thus, the generation of pharmacophore- and shape-based models, which could discriminate between these activity classes, was not successful at this stage.

To gain further insights into the different interaction patterns of agonists and antagonists, we directly docked 45 agonists, 47 antagonists, and 148 inactive compounds into the empty binding pocket of the MOR. Interestingly, we could observe quite substantial predicted interaction pattern differences. The majority of active ligands at the MOR, both agonists and antagonists, were predicted to form a charged interaction with Asp147 and a hydrogen bond with Tyr148 (Fig. 1). Specifically, 36 out of 45 agonists (80.0%) and 42 out of 47 antagonists (89.4%) displayed this binding mode. In contrast, this interaction pattern was only observed for 37 out of the 148 inactive compounds, representing 25.0% of the “inactives” dataset. In addition, we could note that 22 of the 42 antagonists (52.4%) (Fig. 1b) were predicted to form additional polar interactions with other residues such as Lys233, Gln124, Glu229, Gln229, Asn150, Trp318, and Tyr128, while only 11.1% (4 out of 36) agonists did so (Fig. 1a). Furthermore, about two thirds of both agonists and antagonists were predicted to form hydrogen bonds with His297 either directly or *via* a water network. Site-directed mutagenesis studies exchanging the corresponding His291 in the κ opioid receptor (KOR) to Phe revealed that the binding of selected ligands was not abrogated^17^, thereby suggesting that His291 may contribute to ligand binding rather *via* hydrophobic interactions than H-bonding. As one third of active compounds in our dataset did not show this H-bond interaction pattern with His297, we did not consider it as crucial. Additionally, five agonists and three antagonists formed polar interactions with the residue Tyr326 and two agonists also interacted with Met151. However, since these interactions were only found in subsets of the datasets and were more or less equally distributed between both activity groups, they were not included in further investigations.

Based on these findings, we concluded that interactions with the amino acid residues Asp147 and Tyr148 are crucial requirements for ligands to bind to the MOR, and molecules displaying additional polar interactions with other residues are more likely to be antagonists rather than agonists. Fig. 1 shows the predicted binding modes exemplified for two well-known and clinically used opioid drugs from the class of morphinans, the agonist morphine **1** (Fig. 1a) and the antagonist naltrexone **2** (Fig. 1b). The crucial role of Asp147 has been already described by several groups earlier^18^,^19^,^20^,^21^ and is well accepted. However, data concerning the role of Tyr148 is limited. In 1996, molecular modeling studies by Tang *et al.*^22^ proposed that this residue is involved in the binding of ohmefentanyl to the MOR. This prompted Xu *et al.*^23^ to assess the binding affinity of a variety of ligands on a Tyr148Phe mutant. The binding affinity of most investigated ligands was decreased, thereby supporting our current hypothesis. In addition, Befort *et al.*^24^ explored the corresponding residue Tyr129 in the closely related δ opioid receptor (DOR), also demonstrating the important role of this residue in ligand binding.

**Figure 2:**
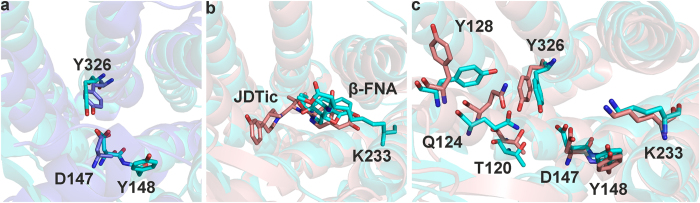
Analysis of the binding sites of MOR, ADRB2, and KOR. (**a**) Alignment of the antagonist β-FNA bound to the MOR (cyan) and the fully activated ADRB2 (blue) shows similar conformations for Asp147 and Tyr326. Tyr148 is absent in the ADRB2. (**b**) A part of the opioid receptor binding pocket is occupied by the KOR-ligand JDTic (salmon), but not by the MOR-ligand β-FNA (cyan), which is covalently attached *via* Lys233. (**c**) Alignment of the MOR (cyan) and the KOR (salmon) revealed conformational changes including Thr120, Gln124, Tyr128, and Tyr326 (numbering according to the MOR).

The MOR is known to undergo a conformational change (also called molecular switch) after activation, which ultimately makes the G protein binding site accessible for its interaction partner^25^. Our results therefore suggest that additional interactions besides Asp147 and Tyr148 may stabilize the receptor in the inactive state. This led us to take a closer examination of the crystal structure, where we assumed that the covalent interaction with Lys233 blocks the proceeding of the conformational change. However, this would also imply that several amino acid residues in the β-FNA-MOR structure did already undergo this structural change, and the complex may therefore rather represent a semi-activated state than an inactive one as originally described^10^. Since for the MOR and other members of the opioid receptor family, the KOR^17^ and the DOR^26^, only the structures in complex with an antagonist have been reported so far, we considered the crystal structure of the β_2_ adrenoreceptor (ADRB2, PDB-entry 3SN6^27^). At this time, it was the only reported GPCR structure that could be solved in an active conformation in complex with the G protein^27^. Indeed, when the MOR residues Asp147 and Tyr326 (Tyr148 is absent in the ADRB2) and the ADRB2 analogues Asp113 and Tyr316 were compared, no differences in their conformation could be observed (Fig. 2a).

The crystal structure of the human KOR in complex with the reversible and selective antagonist JDTic (PDB-entry 4DJH^17^) was reported at the same time as the MOR structure^17^. A comparison of the two structures revealed that JDTic occupies a part of the binding pocket β-FNA does not enter (Fig. 2b). Therefore, we assumed that residues in this area of the binding site (for example Thr120, Gln124, Tyr128, and Tyr326) might be involved in the conformational change. In the KOR structure, they might adopt the inactive conformation, possibly due to steric hindrance by the antagonist, whereas in the MOR structure, they might represent the active conformation. A detailed comparison of the two structures revealed that Asp147 and Tyr326 bended towards another and were located within H-bond formation distance in the MOR. This H-bond cannot be formed in the KOR, because moving of Tyr320 (in KOR) would cause a clash with JDTic. However, this bending of Tyr326 (in MOR) disrupted the direct interaction with Thr120 (corresponding to Thr111 in the KOR) that can be observed in the KOR structure. To compensate for this abrogated direct interaction, Glu124 (MOR) shifted down and mediates an H-bond network between Thr120 and Tyr326. We assumed that the proceeding of the conformational change may be stopped by the covalent interaction of β-FNA with Lys233, and indeed no conformational differences in the MOR and KOR structure could be observed for this residue. Fig. 2c shows the conformational differences observed in the MOR and KOR structures, thereby supporting our hypothesis, that the MOR structure may represent a semi-active state.

Based on the mode of action analysis outcomes, we again aimed to generate pharmacophore- and shape-based models for agonists and antagonists. In the previous attempts, we had focused on the representation of the larger N- substituents (i.e. allyl and cyclopropylmethyl) in the antagonist models. For that purpose, we had included hydrophobic pharmacophore features (Hs) or color features in the pharmacophore and shape-models, respectively, which should be mapped by the antagonists. On the other side, we have tried to prohibit mapping of these compounds in the agonist models by adding exclusion volumes (XVols) and keeping the shape small in the relevant areas. However, after our mode of action analysis, we rather focused on the representation of the interactions that we identified as important for binding and biological activity.

#### Pharmacophore Modeling

**Figure 3:**
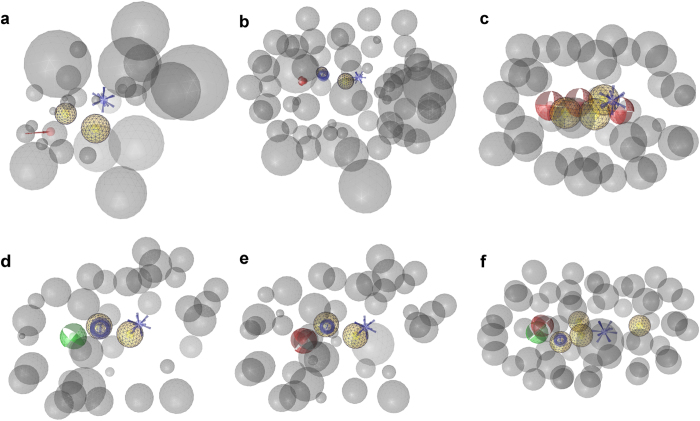
MOR pharmacophore models. (**a**) The structure-based pharmacophore model pm-ag-4dkl-model-13 consisted of one HBA, one PI, two hydrophobic features (Hs), and 29 exclusion volumes (XVols). (**b**) The ligand-based pharmacophore model pm-ag-lig-model-1 contained one HBA, one PI, one aromatic feature (Ar), two Hs, and 62 XVols. (**c**) The ligand-based model pm-ag-lig-model-2 contained three HBAs, one PI, three Hs, and 41 XVols. (**d**) The ligand-based model pm-ant-lig-model-3 consisted of one HBD, one PI, two Hs, one Ar, and 36 XVols. (**e**) The ligand-based model pm-ant-lig-model-4 contained one HBA, one PI, two Hs, one Ar, and 44 XVols. (**f**) The ligand-based model pm-ant-lig-model-5 consisted of one HBD, one HBA, one Ar, four Hs, and 52 XVols. HBA: red sphere or arrow, HBD: green sphere, H: yellow sphere, PI: blue star, Ar: blue ring, XVol: grey sphere.

In total, the three agonist (Fig. 3a–c) and antagonist models (Fig. 3d–f) that performed best during the theoretical validation and facilitated the highest recovery of active molecules from the dataset were employed for virtual screening. The performances of the individual models and the structural data that was used for model generation is summarized in Table 1. A detailed description of the model generation and the agonist and antagonist training compounds S1–S5 and S6–S10 is provided in Supplementary Section S1 and Supplementary Charts S1 and S2, respectively, of the Supporting Information.

All three agonist models together covered 39 out of the 45 agonists (86.7%), but only mapped 8 antagonists (17.7%) and one out of 148 inactive compounds (0.7%). In combination, they retrieved an area under the curve (AUC) in the ROC-plot of 0.89 and an enrichment factor (EF) of 4.3 (81.0% of the maximum (max) EF).

The three antagonist models together mapped 42 out of the 47 antagonists in the dataset (89.4%), but also 30 of the 45 agonists (66.7%) and 5 inactive compounds (3.4%). They yielded an AUC of 0.85, and an EF of 2.8, which is 54.9% of the maxEF. However, if also the agonists in the dataset are considered as “active”, than the performance of the models improves with an AUC of 0.88 and an EF of 2.4 (92.3% of the maxEF). The MOR antagonist models do not retrieve many inactive compounds, but they fail to efficiently discriminate between agonists and antagonists. As they matched also several agonists, they may rather be considered as general MOR ligand models than as selective MOR antagonist models.

#### Shape-based modeling

In total, 68 shape-based MOR agonist and 32 antagonist models were created. Similar to pharmacophore modeling, only the best performing ones during the theoretical validation were selected for prospective virtual screening. An overview of the models and their performances is provided in Table 1. The shape-based model generation (Supplementary Section S2) and the agonist (Supplementary Chart S3) and antagonist (Supplementary Chart S4) training set is described in detail in the Supporting Information.

**Figure 4:**
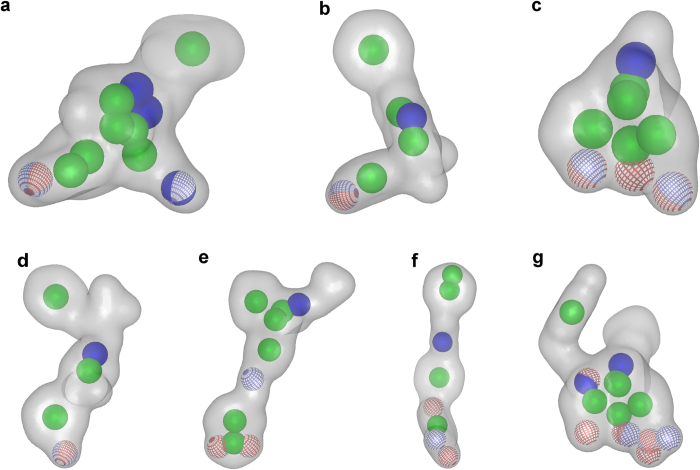
Shape-based MOR models. (**a**) The ligand-based model shape-ag-model-1 was created with one aligned low-energy conformation of 7-aminomorphan derivative **S10** and phenazocine derivative **S3**. (**b**) The ligand-based model shape-ag-model-2 was generated with one low-energy conformation of the agonist quinolizidine derivative **S11**. (**c**) The ligand-based model shape-ag-model-3 was built with one low-energy conformation of morphine **1**. (**d**) The model shape-ant-model-1 was created with one low energy conformation of alvimopan **S7**. (**e**) The MOR antagonist MCL-702 **S12** served as query molecule for the second antagonist model shape-ant-model-2. (**f**) The model shape-ant-model-3 was based on one low-energy conformation of the antagonist **S13**. (**g**) The model shape-ant-model-4 was generated with one aligned conformation of the MOR antagonists ALKS-33 **S14** and **S15**. HBA: Red spheres mesh, HBD: blue spheres mesh, cation: blue spheres solid, ring: green spheres solid.

All three selected agonist models (Fig. 4a–c) together identified 32 out of the 45 agonists (71.1%), 13 of the 47 antagonists (27.6%), and 3 out of 148 inactives (2.0%). This led to an AUC of 0.82 and an EF of 3.6, representing 66.7% of the maxEF.

When the four antagonist models were combined (Fig. 4d–g), they mapped 39 out of the 47 antagonists (83.0%), 14 agonists (31.1%), and one inactive compounds (0.7%). All models together therefore retrieved an AUC of 0.88 and an EF of 3.7 (72.3% of the maxEF).

#### Selection of exemplary virtual hits for biological testing

To investigate whether the generated models and the docking protocol are able to identify novel active ligands for the MOR, all theoretically validated pharmacophore- and shape-based models and the docking protocol were used for virtual screening using the commercial Maybridge database. Following virtual screening, exemplary virtual hits from every method and for every activity class were selected for biological testing. In detail, the top-four ranked molecules from the agonist- and the top-two ranked molecules from the antagonist screening were selected. An overview of the pharmacophore- and shape-based virtual screening is provided in Supplementary Fig. S1 in the Supporting Information. All selected test compounds are listed in Table 2.

### Pharmacology

#### *In vitro* pharmacology

**Figure 5:**
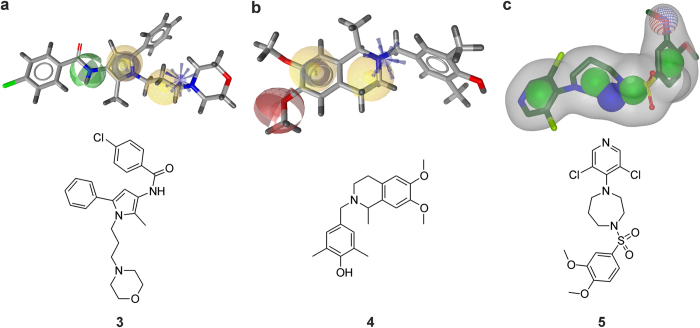
The active compounds are aligned to the mapping models. Compounds **3** (**a**), **4** (**b**), and **5** (**c**) map model pm-ant-lig-model-3 (**a**), pm-lig-ant-model-4 (**b**), and shape-ag-model-2 (**c**), respectively.

In the hunt for the identification of novel scaffolds as ligands at the MOR, the 18 compounds selected from the virtual screening hits (from i.e. pharmacophore modeling, shape-based modeling, and docking) were purchased for experimental testing. The primary biological screen consisted of a competitive inhibition binding assay at the human MOR. The capability of the 18 compounds and the well-established reference MOR ligand, morphine **1**, all tested at a concentration of 10 μM, to inhibit binding of the selective MOR radioligand [^3^H][D-Ala^2^, Me-Phe^4^, Glyol^5^]enkephalin ([^3^H]DAMGO) was assessed with membranes from Chinese hamster ovary cells stably transfected with the human MOR (CHO-hMOR)^28^ (Supporting Information, Supplementary Fig. S2). Among the tested hits, three molecules **3**, **4**, and **5** inhibited by ≥50% [^3^H]DAMGO binding to the MOR, and their properties were therefore further investigated. Their structures are presented in Fig. 5. The structures of the virtual hits that were inactive in the biological testing are provided in Supplementary Table S1.

**Figure 6:**
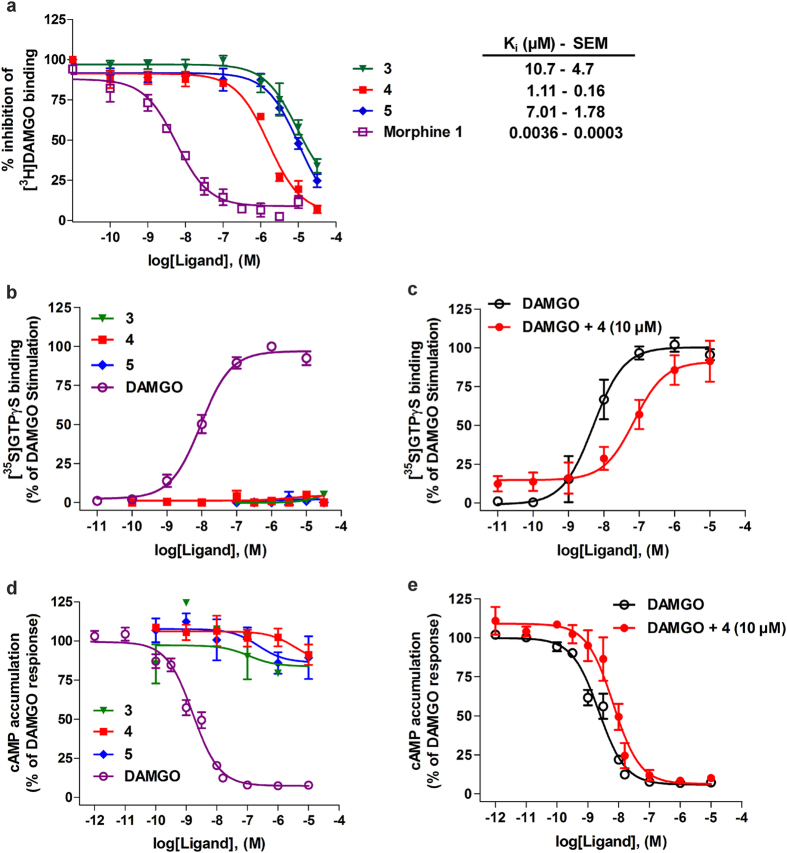
*In vitro* pharmacological profile of compounds 3, 4, and 5 on the human MOR. (**a**) Competitive inhibition of [^3^H]DAMGO binding in membranes from CHO-hMOR cells. Binding affinities as K_i_ values were calculated from the competitions binding curves. (**b**) Stimulation of [^35^S]GTPγS binding in membranes from CHO-hMOR cells. (**c**) Effect of **4** on DAMGO-stimulated [^35^S]GTPγS binding. (**d**) cAMP accumulation inhibition in CHO cells co-expressing human MOR and the cAMP biosensor. (**e**) Effect of **4** on DAMGO inhibition of forskolin-stimulated cAMP accumulation. Experimental data were analyzed and graphically processed using the GraphPad Prism Software. All values are expressed as the mean ± SEM (n = 3). Nonvisible SEM is within the symbol.

The new ligands **3**, **4**, and **5** were next tested in concentration-dependent competition binding studies to quantify their affinities at the MOR in CHO-hMOR cell membranes. Competitive inhibition of [^3^H]DAMGO binding to the MOR in a concentration-dependent manner was shown by all three molecules, with **4** identified as the most active ligand based on the inhibition constant (K_i_) value of 1.11 μM, albeit considerably lower than that of morphine **1** (Fig. 6a). To assess whether **3**, **4**, and **5** behave as MOR agonists or antagonists, we used two functional assays that measure G protein-mediated [^35^S]GTPγS binding^28^ and cAMP production^19^. As shown in Fig. 6b, none of the three ligands produced any increase in the [^35^S]GTPγS binding in membranes from CHO-hMOR cells, consequently indicating an antagonist profile at the MOR, in contrast to the high potency and stimulatory effect shown by the prototypical MOR agonist DAMGO. Additional investigations with the most interesting ligand **4** established its MOR antagonism, based on the14-fold rightward shift in the DAMGO concentration-response curve in the presence of 10 μM of **4** (Fig. 6c), thus giving an antagonist equilibrium constant (K_e_) of 1.02 ± 0.25 μM. A similar profile was observed when performing the cAMP accumulation assay, a cell-based assay, nowadays extensively applied in the drug discovery process for a large number of seven transmembrane-GPCRs to investigate post-receptor G protein-mediated responses by measuring changes in cAMP levels^29^,^30^. While in CHO cells co-expressing the human MOR and the cAMP biosensor GloSensor-22F (CHO-hMOR-p22F cells), the agonist DAMGO produced a concentration-dependent inhibition of the forskolin-stimulated cAMP production displaying high potency, **3**, **4**, and **5** were not effective in this assay (Fig. 6d). In the MOR-cAMP assay, **4** acted as an antagonist affording a 3-fold rightward shift in the concentration-response for DAMGO, and a K_e_ of 8.03 ± 0.38 μM for **4** (Fig. 6e).

#### *In vivo* pharmacology

**Figure 7:**
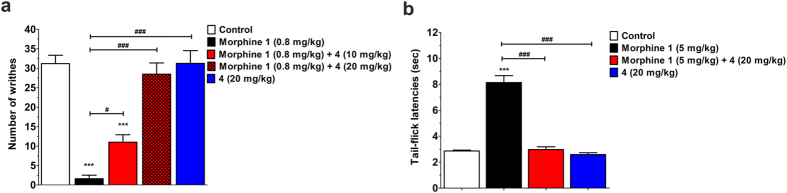
*In vivo* MOR antagonist activity of compound 4. Antagonism of morphine-induced antinociception in mice by **4** after s.c. administration in (**a**) the acetic acid-induced writing test and (**b**) the tail-flick test. Compound **4** was administered 30 min prior to s.c. morphine, and number of writhes or tail-flick latencies were determined 30 min after morphine administration. ***p < 0.001 *vs.* control (vehicle) group; ^#^*p* < 0.05 and ^###^*p* < 0.001 *vs.* morphine-treated group. Experimental data were analyzed and graphically processed using the GraphPad Prism Software. All values are expressed as the mean ± SEM (n = 5–6 mice). Nonvisible SEM is within the symbol.

The MOR antagonist activity of compound **4** was further evaluated *in vivo* in mouse models of chemical (acetic acid-induced writhing test) and thermal nociception (tail-flick test), according to the described procedures^28^,^31^. Compound **4**, administered subcutaneously (s.c.) to mice, was investigated for its ability to antagonize s.c. morphine-induced antinociception in both pain tests (Fig. 7). When **4** was given 30 min prior to morphine, a significant and dose-dependent reversal of morphine analgesia in the writhing test was measured (Fig. 7a). Pretreatment of mice with a dose of 20 mg/kg of **4** also elicited a complete blockade of morphine’s analgesic effect against thermal-induced nociception (Fig. 7b). Moreover, compound **4** administered alone did not affect pain behavior, as there were no alterations in chemical or thermal sensitivity of animals receiving s.c. **4**, when compared to control (vehicle) treated mice.

## Discussion

Over the years, the MOR has received extensive attention as a prominent drug discovery target due to its central role in mediating a variety of neurophysiological and behavioral responses, including pain, reward and addictive behavior, and gastrointestinal motility^1^,^2^. Thus, there has been constant focus on the discovery of innovative ligands at the MOR with potential for their development as new and safer therapeutics^2^,^3^,^4^. The crystal structure of the murine MOR available today^10^ makes it feasible to analyze the ligand-receptor and structure-function relationships of the human MOR, as the ultimate target of therapeutic opioid drugs.

Herein, we present the outcomes of application and combination of molecular modeling and pharmacological approaches for the discovery of novel ligands as agonists or antagonists at the MOR. A detailed evaluation and discussion of the applied virtual screening tools is provided in Supplementary Section S3 in the Supporting Information. As a result of different computational approaches, two active ligands at the MOR, **3** and **4**, were identified by pharmacophore-based virtual screening and one molecule, **5**, was identified with shape-based virtual screening. The three active compounds discovered in the current study had low binding affinities to the MOR *in vitro*, combined with MOR antagonist properties. *In vivo*, however, compound **4** was highly effective in antagonizing morphine-induced antinociception in mice. Notably, in contrast to many important and well-known MOR ligands, these compounds represent novel chemical scaffolds. It is commonly recognized that hits identified in a virtual screening campaign often display weaker activity than the compounds the models were based on^32^. Actually, if we applied our own activity cut-off of 1000 nM as used for the inactives dataset, we would have to consider all of the tested compounds as inactive. However, a rather narrow activity definition profile was selected for modeling in order to increase the probability of discovering “higher” active compounds. Additionally, if molecules with binding affinity at the MOR (K_i_ or IC_50_ value) of 1000 nM were included, then we would have also trained the models to find weaker active compounds *per se*, thereby risking the mapping of even less active ligands.

Intriguingly, all novel MOR ligands identified in this study display the activity of antagonists. Despite the many agonists mapping the antagonist pharmacophore models in the theoretical validation, both proved to prioritize antagonists in the prospective screening. Compound **5**, however, was identified with an agonist shape-model, thereby incorrectly predicting its activity. Additionally, we targeted the novelty of the identified ligands. To this aim, we compared them to the 92 known active molecules in the actives dataset (45 agonists and 47 antagonists) using the “Compare Libraries” tool implemented in Discovery Studio^33^. This tool calculates the most similar compounds in the actives dataset compared to the novel ligand using extended connectivity fingerprints 4 (ECFP4) and the Tanimoto coefficient (Tc)^34^,^35^. The Tc ranks from zero to one, where a score of one is retrieved for identical compounds, and a very low score indicates structurally distinct molecules. The analysis revealed that the most similar compounds in the active datasets had a Tc of 0.17284 for 5, 0.22973 for **4**, and 0.21519 for **3**. The overall similarity of the compounds in comparison to the total dataset was calculated to be 0.0263 for **5**, 0.0301 for **4**, and 0.0390 for **3**. In addition, a detailed literature search concerning the novelty of the identified compounds was performed using SciFinder. No reference associated compounds **3 - 5** with the MOR or any other opioid receptor. For compounds **3** and **5**, also no close analogues with a 2D similarity of Tc ≥ 80 have been connected to opioid receptors so far. However, a limited number of derivatives of compound **4** have been earlier investigated for MOR activity. Laudanosine was reported to lower radioligand binding to μ1 and μ2 receptors with K_i_ values of 2.7 μM and 13 μM, respectively, and to exert antinociceptive effects in the mouse tail-flick test^36^, indicative for an agonist profile. Several natural morphine precursors have been tested for MOR activity as well, however, all of them were inactive^37^. The most active close analogue of **4**, reticuline, showed ca. 16% of morphine effects in a FRET cell-based assay at a concentration of 100 μM^37^. In addition, no binding of reticuline to the μ3 opioid receptor subtype could be observed in human monocytes^38^. Compound **4** is therefore the only member of this chemical class that has been reported to display an antagonistic activity up to now. Several other analogues of **4** have been isolated from various *Berberis* species, however, no information is available concerning their activities at the MOR. We investigated nummularine^39^, bernumidine, bernumicine^40^, and bernumine^41^, amenurine, and *O*-acetylamenurine^42^ within our docking workflow, respectively. Our results predicted an agonist MOR activity for bernumine, amenurine, and *O*-acetylamenurine. In addition, nummularine was predicted as antagonist. Several studies reported biological effects of *Berberis vulgaris* extracts that could be mediated *via* the MOR^43^,^44^,^45^. However, to date no constituents similar to **4** have been isolated from this species. Further investigations are therefore required to elucidate the mechanisms underlying the effects of *Berberis vulgaris* extracts and to identify the active constituents. Additionally, *Berberis nummularia*^39^,^40^,^41^ and *Berberis amurensis*^42^ containing nummularine^39^, bernumidine, bernumicine^40^, bernumine^41^, amenurine, and *O*-acetylamenurine^42^ would be particularly interesting for further investigations. Thus, despite the low binding affinity at the MOR, the molecules identified as MOR ligands in this study represent an interesting starting point for further chemical optimization due to their structural novelty.

The detailed analysis of the docking poses of agonists, antagonists, and inactive compounds allowed for further insights into the mode of action. This mode of action remains hypothetical as there is no experimental data available that could support our present concept. However, this was beyond the scope of the study presented herein. In the course of preparation of this report, a novel crystal structure of the MOR in complex with the morphinan BU72 was published (PDB entry 5C1M)^46^, thereby providing first insights into the binding mode of morphinan agonists. In the reported structure, BU72 interacts with the MOR *via* the crucial charged interaction with Asp147 and the hydrogen bond, although water-mediated, to Tyr148. Besides various hydrophobic interactions, which were however not in the focus of this study, it was also found to form a hydrogen bonds to His54 and, water-mediated, to the backbone of Lys233. At first sight, this observation may be in contrast to our own original hypothesis. However, His54 is part of a flexible loop which is not present in the 4DKL structure, thus prohibiting us to study its influence. More important, mutations of His54 did not alter BU72 binding or receptor activation, suggesting that this interaction does only play a minor role. Also, the water-mediated interaction with the Lys233 backbone is in line with our current hypothesis, since it still allows for the movement of the side chain (whereas our docking results suggested interactions directly with the side chain of Lys233, thereby inhibiting any shifts), and a direct comparison of this residue in the 4DKL and the 5C1M structure revealed a different position of Lys233 in the active MOR structure (Supplementary Fig. S3), showing that the binding of BU72 did allow for conformational changes of Lys233. Although these conformational re-arrangements appeared to be only subtle for Lys233, it provoked pronounced conformational adaptions similar to a chain reaction in neighboring residues interacting *via* a polar network (Supplementary Fig. S3). Intriguingly, the parts of the binding pocket, which we assumed to be already in the active state, do not differ substantially from the 4DKL structure (except Tyr128 as shown in Supplementary Fig. S4), and the overall binding site of 4DKL closely resembled the 5C1M structure after molecular dynamics simulation^46^. This novel structural data strongly supports our hypothesis as (I) the agonist forms interactions which we identified as crucial, (II) structural differences in the binding pockets of the two structures are subtle, suggesting that the 4DKL structure represents indeed a semi-active state, and (III) conformational changes caused by and including Lys233 point towards this residue in blocking the proceeding of the conformational change in the 4DKL structure.

Recently, Leonis *et al.*^47^ investigated the closely related opioid receptor, KOR, by applying a molecular dynamics simulation approach. Analogous to our results, they could also observe that the KOR agonist salvinorin A (SalA) was involved in less polar interactions, and the whole complex appeared to be more flexible as compared to the antagonist JDTic-KOR complex^47^. The role of the key amino acid residues Asp147 and Tyr148 identified in our study appears to be controversial in the Leonis-study on the KOR. The corresponding residues in the KOR, Asp138 and Tyr139, appeared to be important for the binding of the antagonist JDTic to the KOR in water, whereas the hydrogen bond to Tyr139 was abolished in the simulation performed in a lipid bilayer. In contrary, hydrogen bond formation of the agonist SalA and Tyr139 could only be observed in the latter setting. The structure of SalA does not contain any nitrogen, and the authors concluded that Asp138 might therefore even display negative effects on the binding of this ligand^47^. During the preparation of this manuscript, Li *et al.*^48^ reported the predicted structures of the KOR bound to five agonists. All of these compounds were found to form polar interactions with Asp138 and in addition also with His291 (corresponding to Asp147 and His297 in the MOR)^48^. The results of Li *et al.* suggest a crucial role for His291/His297 in the binding of ligands. In both the 4DKL and the 5C1M structure water-mediated hydrogen bonds with this residue were observed and also a subset of active compounds, both agonists and antagonists, were found to interact with His297 in our study. However, this did not apply for about one third of active compounds in our dataset. In addition, ligand binding in a His291Phe mutant of the KOR was reported to be unaffected^17^. The elucidation of the role of His297 in ligand binding and receptor activation therefore still requires further investigations. All agonists including the two morphinan compounds investigated by Li and colleagues^48^ were located in close proximity to Tyr139 (corresponding to Tyr148 in the MOR), however, other than in our current study, no hydrogen bond formation was observed. This is in contrast to the three experimentally confirmed morphinan-opioid receptor structures, where β-FNA, BU72, and naltrindole formed H-bonds with Tyr148 in the MOR and the corresponding Tyr129 in the DOR. Also, JDTic potentially interacts with Tyr139 in the KOR crystal structure *via* a water network. When Wu *et al.*^17^ reported the crystal structure of the KOR, they also docked two selective antagonists, nor-binaltorphimine and 5′-guanidinonaltrindole, into the binding pocket of the KOR. Intriguingly, these two ligands were also reported to form additional polar interactions with amino acid residues other than Asp128 and Tyr129^17^.

The comparison of the β-FNA-MOR and the JDTic-KOR complexes revealed distinct binding poses of the two ligands, whereas the crystal structure of the DOR in complex with the selective antagonist naltrindole (PDB entry 4EJ4^26^) shows similar interaction patterns. However, both naltrindole and β-FNA have a morphinan structure, in contrast to the non-morphinan ligand JDTic. The docking protocol presented in this study was optimized for the binding site of the co-crystallized ligand, so it might be less suitable for molecules displaying other binding modes, which might be relevant e.g. for some other non-morphinans. In addition, ligands occupying the side pocket similar as JDTic in the KOR structure may not be correctly fitted into the binding pocket, as parts of the conformational change they would prohibit has already occurred in 4DKL structure (Fig. 2c). More structural information, especially of non-morphinan MOR complexes, may therefore be required to further address this issue.

## Conclusions

By using an application and combination of molecular modeling and pharmacological approaches, novel MOR ligands were successfully identified in the present study. Although they interact with the receptor relatively weakly, the new chemotypes showed MOR antagonist properties representing valuable starting points for further chemical optimization, development into neurochemical tools for studying the MOR, as well as the potential of developing therapeutic lead candidates by providing innovative prospects for rational opioid drug design. Our initial findings indicate that the generated models and workflows have a promising potential for the identification of novel scaffolds as ligands at the MOR.

Moreover, the application of selected common virtual screening tools herein allowed for gaining significant insights into the distinct interaction patterns of MOR ligands. Although the presented concept remains hypothetical, it substantially aid to the development of virtual screening workflows that successfully discovered novel bioactive molecules at the MOR, and the novel structural data on the MOR strongly supports our findings. The crystal structure of the MOR in complex with an irreversible antagonist, β-FNA, supports the elucidation of the mode of action. However, structural data concerning the MOR, preferably in complex with a non-morphinan ligand, are still required to gain understandings into the molecular mechanism underlying the different biological activities observed upon binding of different structurally diverse ligands to the MOR.

Overall, the present results provide significant support that the appropriate selection of computational methodologies represents a viable approach towards discovery of active molecules with new chemical scaffolds interacting with the MOR. Furthermore, understanding the ligand-receptor interactions and structure-function relationships of the MOR are essential steps towards the development of improved pharmacotherapies for neurological and other disorders, where the MOR plays a key role.

## Methods

### Study Design

**Figure 8:**
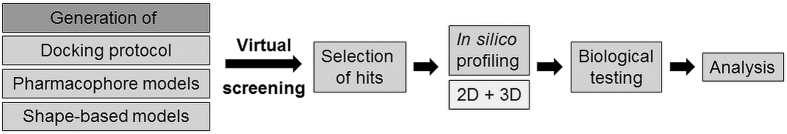
Study design.

An overview over the study design is depicted in Fig. 8. A docking workflow that could retrospectively discriminate between agonists and antagonists was established. In addition, pharmacophore and shape-based models were generated separately for both MOR agonists and antagonists. After theoretical validation, all the generated models and the docking protocol were used for virtual screening of the Maybridge database (www.maybridge.com). Exemplary top-ranked agonist and antagonist hits of all methods were selected for further investigations with the external profiling tools SEA^49^, PASS^50^, PharmMapper^51^, and PharmaDB^52^, (these results are provided in Supplementary Section S3 in the Supporting Information) and subsequently subjected to biological evaluation. Compounds that were active in a first screening were investigated further. The results of the theoretical validation and the experimental assessment were used to evaluate the performances of all applied methods (A detailed discussion of the performances of the applied tools is provided in Supplementary Section S4 of the Supporting Information).

### Molecular modeling

#### Prospective virtual screening

All pharmacophore and shape-based modeling studies, the docking studies, and the virtual screening were performed with LigandScout version 3.1^12^, vROCS version 3.0.0^13^,^14^, and GOLD version 5.2, respectiely^15^. A detailed description of these programs and the bioactivity profiling tools is provided Supplementary Section S5 of the Supporting Information.

#### Dataset

For the model generation, optimization, and theoretical validation, known active ligands were manually assembled from the original literature. Ligands were considered to be active based on the MOR binding affinity, defined as K_i_ or IC_50_ (in case of ligand-displacement assays) values of ≤5 nM. In addition, ligands had to be confirmed full agonists or antagonists. This led to 45 agonists and 47 antagonists in the datasets. The vast majority of MOR ligands had a morphinan structure, however, we aimed to keep the dataset as diverse and small as possible. Therefore, not all active morphinans were included in the dataset, even if more compounds are reported in public repositories such as the ChEMBL^53^. The structures of the compounds in the “agonist” and “antagonist” dataset are provided in Supplementary Tables S2 and S3 of the Supporting Information. For the “inactives” dataset, 148 compounds with a K_i_ or IC_50_ value of ≥1000 nM were collected. The majority of inactive compounds (90.5% of the dataset) contain at least one nitrogen, which would be in principle a basic requirement for interacting with Asp147. We applied a rather strict cut-off for inactive molecules, because virtual hits are usually less active than the compounds that were used for building the models^32^. The smiles codes of the inactive compounds can be found in Supplementary Table S4. The commercial Maybridge_HitDiscover database (www.maybridge.com, access date 27. February 2014), which contains about 52,000 diverse molecules, was used for the prospective virtual screening.

#### Theoretical model validation

All newly generated models were carefully validated using the “actives” and “inactives” datasets. For assessment of a models quality, the EF was calculated using Equation1, where TP represents the number of true positive hits, n the total number of virtual hits, A the number of active compounds in the database, and N the total number of compounds in the database.


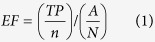


The EF calculates the enrichment of active compounds in the virtual hitlist compared to a random selection of compounds. However, the EF is highly dependent on the composition of the dataset. Therefore, we also calculated the % of the maxEF a model yielded. Whenever models were applied together, the AUC^54^ of the ROC plot was calculated as additional quality assessment of our model collection.

### *In vitro* pharmacology

#### Chemicals and materials

[^3^H]DAMGO and [^35^S]GTPγS were purchased from PerkinElmer (Boston, USA). DAMGO, naloxone, dimethylsulfoxide (DMSO), tris(hydroxymethyl)aminomethane (Tris), 2-[4-(2-hydroxyethyl)piperazin-1-yl]ethanesulfonic acid (HEPES), GTPγS, guanosine diphosphate (GDP), forskolin and Hanks’ balanced salt solution (HBSS) were obtained from Sigma-Aldrich Chemicals (St. Louis, MO). Morphine hydrochloride was obtained from Gatt-Koller GmbH (Innsbruck, Austria). All cell culture media and supplements were from Sigma-Aldrich Chemicals (St. Louis, MO) and Invitrogen (Paisley, UK). All other chemicals were of analytical grade, and were obtained from standard commercial sources. The selected compounds for biological testing were obtained from Maybridge (www.Maybridge.com), and their purities were all higher than 95%.

#### Cell culture

CHO cells expressing the human MOR (CHO-hMOR cells), a gift from Dr. Lawrence Toll (SRI international, Menlo Park, CA), were grown in Dulbecco’s modified Eagle’s medium (DMEM)/Ham F-12 medium supplemented with fetal bovine serum (10%), penicillin/streptomycin (0.1%), L-glutamine (2 mM) and geneticin (400 μg/mL). CHO-hMOR cells were stably transfected with the cAMP biosensor GloSensor-22F (Promega, Madison, WI), a modified form of firefly luciferase containing a cAMP-binding motif. Transfection was performed using the Viafect^TM^ transfection reagent (Promega), according to the manufacturer’s instructions, and positive clones were selected with hygromycin B (400 μg/mL). All cell cultures were maintained at 37 °C in 5% CO_2_ humidified air.

#### Radioligand MOR binding assay

Ligand binding experiments were carried out with the specific MOR radioligand [^3^H]DAMGO using membranes prepared from CHO-hMOR cells, according to the previously described procedure^28^. Cell membranes (15 μg protein) were incubated with [^3^H]DAMGO (1 nM) and various concentrations of the test compound in 50 mM Tris-HCl buffer (pH 7.4) for 60 min at 25 °C in a final volume of 1 mL. Nonspecific binding was defined in the presence of 10 μM naloxone. Samples were filtered through Whatman GF/C glass fiber filters, and rinsed with ice-cold 50 mM Tris-HCl buffer (pH 7.4). Radioactivity retained on the filters was measured by liquid scintillation counting. Each binding experiment was performed in duplicate and repeated at least three times. K_i_ values were calculated by the Cheng and Prusoff equation^55^, using the K_d_ value of 1.59 nM for [^3^H]DAMGO determined from the saturation binding curves.

#### [^35^S]GTPγS binding assay

Determination of [^35^S]GTPγS binding to CHO-hMOR cell membranes were performed as described previously^28^. Cell membranes (5–10 μg protein), prepared as described for the radioligand MOR binding assay, were incubated in the absence or in the presence of test compound in the assay buffer (20 mM HEPES, 100 mM NaCl, 10 mM MgCl_2_, pH 7.4) containing [^35^S]GTPγS (0.05 nM) and GDP (10 nM) in a total volume of 1.0 mL for 60 min at 25 °C. Nonspecific binding was determined using 10 μM GTPγS, and the basal binding was determined in the absence of test compound. Samples were filtered over glass fiber filters and counted as described for the radioligand binding assays. The enhancement of [^35^S]GTPγS binding above the basal activity was used to determine the potency (EC_50_) and efficacy (E_max_, as percentage of maximum stimulation with respect to the reference MOR agonist, DAMGO, which was set as 100%). To determine the MOR antagonist activity of **4**, a concentration-response curve for DAMGO was obtained by assessing the [^35^S]GTPγS binding to CHO-hMOR cell membranes in the presence or absence of 10 μM of **4**, as previously described^56^. The ratio of EC_50_ values of DAMGO in the presence and absence of **4** was determined to provide the dose ratio (DR). The antagonist equilibrium dissociation constant K_e_ for **4** was calculated using the equation K_e_ = [a]/(DR − 1), where “a” is the concentration of antagonist. All experiments were performed in duplicate and repeated at least three times.

#### cAMP accumulation assay

Inhibition of the forskolin-stimulated intracellular cAMP accumulation in CHO cells co-expressing the hMOR and the cAMP biosensor GloSensor-22F (CHO-hMOR-p22F cells) was performed using the Glo-Sensor^TM^ cAMP assay (Promega), according to the manufacturer’s instructions. Cells were seeded in growth medium into 384-well plates at a density of 5,000 cells in 30 μL per well and incubated overnight. On the day of assay, culture media was removed, and cells were pre-equilibrated for 90 min with 4% v/v of the GloSensor cAMP reagent in reaction medium (20 mM HEPES, 1 × HBSS, pH 7.4) at 37 °C and 5% CO_2_. Cells were then treated with various concentrations of the test compounds or the reference MOR agonist DAMGO, for 15 min at room temperature. Forskolin (10 μM) was added to each well, and luminescence was measured after 20 min. All experiments were performed in triplicate and repeated at least three times.

#### Data analysis

Experimental data were analyzed and graphically processed using the GraphPad Prism Software (GraphPad Prism Software Inc., San Diego, CA, USA). Data are expressed as means ± SEM.

### *In vivo* pharmacology

#### Animals and drug administration

Male CD1 mice (30–35 g) were obtained from Charles River (Sulzfeld, Germany). Mice were group-housed in a temperature controlled room with a 12 h light/dark cycle and with free access to food and water. All animal studies were conducted in accordance with ethical guidelines and animal welfare standards according to Austrian regulations for animal research, and were approved by the Committee of Animal Care of the Austrian Federal Ministry of Science and Research. Morphine was dissolved in sterile physiological saline (0.9%). Compound **4** was prepared in 2% DMSO in sterile physiological saline (0.9%). Vehicle or solutions of test compounds were administered s.c. in a volume of 10 μl per 1 g body weight. Each experimental group included five to six animals.

#### Acetic acid-induced writhing test

Writhing was induced in mice by intraperitoneal (i.p.) injection of a 0.6% acetic acid aqueous solution^31^. Drugs or control (vehicle) were s.c. administered, and then 5 min prior to testing (25 min after drug or vehicle) each animal received i.p. acetic acid solution. Each mouse was placed in individual transparent Plexiglas chambers, and the number of writhes was counted during a 10 min observation period.

#### Tail-flick test

The radiant heat tail-flick test was performed using an UB 37360 Ugo Basile analgesiometer (Ugo Basile s.r.l., Varese, Italy)^28^. The tail-flick latencies were measured before and 30 min after drug or control s.c. administration. In the antagonist study, compound **4** was s.c. administered 30 min prior to morphine. A cut-off time of 10 s was used in order to minimize tissue damage.

#### Data analysis

Data were analyzed with ANOVA using Turkey’s for multiple comparisons as *post hoc* test, and graphically processed with the GraphPad Prism Software. Results were considered statistically significant if *p* < 0.05.

## Additional Information

**How to cite this article**: Kaserer, T. *et al.* µ Opioid receptor: novel antagonists and structural modeling. *Sci. Rep.* **6**, 21548; doi: 10.1038/srep21548 (2016).

## Supplementary Material

Supplementary Information

## Figures and Tables

**Table 1:** Quality metrics of MOR agonist and antagonist pharmacophore and shape-based models.

Model	Based on	ComboScore Cut-off	EF	maxEF
Agonist				
Pharmacophore				
pm-ag-4dkl-model-13	PDB-entry 4DKL^10^		5.3	100.0%
pm-ag-lig-model-1	morphinan MOR agonists **S1**^6^ and **S2**^8^ and phenazocine derivative **S3**^57^		4.3	81.0%
pm-ag-lig-model-2	oxymorphone analogues **S4**^6^ and **S5**^4^		3.7	69.6%
Shape-based				
Shape-ag-model-1	7-aminomorphan derivative **S10**^58^ and phenazocine derivative **S3**^57^	≥1.02	4.0	75.5%
Shape-ag-model-2	quinolizidine derivative **S11**^59^	≥1.35	4.3	81.0%
Shape-ag-model-3	morphine **1**^60^	≥1.50	3.4	63.3%
Antagonist				
Pharmacophore				
pm-ant-lig-model-3	naltrexone **2**^61^, the piperidine derivative **S6**^62^, and alvimopan **S7**^63^		3.2	62.7%
pm-ant-lig-model-4	naltrexone **1**^61^, the piperidine derivative **S6**^62^, and alvimopan **S7**^63^		3.5	68.6%
pm-ant-lig-model-5	quinolizidine derivative **S8**^59^ and pyrazine derivative **S9**^64^		5.1	100.0%
Shape-based				
Shape-ant-model-1	alvimopan **S7**	≥1.35	4.1	80.1%
Shape-ant-model-2	MCL-702 **S12**^65^	≥1.20	4.4	85.8%
Shape-ant-model-3	carboxamido biaryl ether derivative **S13**^66^	≥1.65	5.1	100.0%
Shape-ag-model-4	naltrexone derivatives ALKS-33 **S14**^67^ and **S15**^68^	≥1.20	3.5	68.3%

**Table 2:** Selected virtual hits for biological testing.

Cpd.	Method	Predicted activity	Fitness score	Ranking in the virtual hit list	Comment
T1	pharmacophore	agonist	0.9609	1	pm-ag-lig-model-1
T2	pharmacophore	agonist	0.9541	2	pm-ag-lig-model-2
T3	pharmacophore	agonist	0.9538	3	pm-ag-lig-model-1
T4	pharmacophore	agonist	0.9536	4	pm-ag-4dkl-model-13
3	pharmacophore	antagonist	0.955	1	pm-ant-lig-model-3
4	pharmacophore	antagonist	0.954	2	pm-ant-lig-model-4
T5	shape-based	agonist	1.06	1	shape-ag-model-2
5	shape-based	agonist	1.04	2	shape-ag-model-2
T6	shape-based	agonist	1.03	3	shape-ag-model-2
T7	shape-based	agonist	1.01	4	shape-ag-model-2
T8	shape-based	antagonist	1.162	1	shape-ant-model-2
T9	shape-based	antagonist	1.158	2	shape-ant-model-2
T10	docking	agonist	70.9	1	crucial interactions with Asp147 and Tyr148
T11	docking	agonist	69.9	2	crucial interactions with Asp147 and Tyr148
T12	docking	agonist	69.1	3	crucial interactions with Asp147 and Tyr148
T13	docking	agonist	67.5	4	crucial interactions with Asp147 and Tyr148
T14	docking	antagonist	70.8	1	additional interactions with Glu229, Lys233, and Lys303
T15	docking	antagonist	66.5	2	additional interactions with Lys233
