# Supplementary material for: μ Opioid receptor: novel antagonists and structural modeling
Source: Sci Rep. 2016 Feb 18;6:21548. doi: 10.1038/srep21548 (PMC4757823; doi:10.1038/srep21548)
Supplement: Supplementary Information [file srep21548-s1.pdf]

Supporting information

μ Opioid receptor: novel antagonists and structural modeling

Teresa Kaserer[1], Aquilino Lantero[2], Helmut Schmidhammer[2], Mariana Spetea[2]*, Daniela Schuster[1]*

[1]Computer-Aided Molecular Design Group and [2]Opioid Research Group, Department of Pharmaceutical Chemistry, Institute of Pharmacy and Center for Molecular Biosciences Innsbruck (CMBI), University of Innsbruck, Innrain 80-82, 6020 Innsbruck, Austria

*Corresponding authors:

Daniela.Schuster@uibk.ac.at

Mariana.Spetea@uibk.ac.at

Supplementary Section S1. Detailed description of pharmacophore model generation (page S3).

Supplementary Chart S1. Training set MOR ligands for the agonist pharmacophore models and their binding affinities (as K_i) at the MOR (page S5).

Supplementary Chart S2. Training set MOR ligands for the antagonist pharmacophore models and their binding affinity (as K_i) at the MOR (page S6).

Supplementary Section S2. Detailed description of shape-based model generation (page S7).

Supplementary Chart S3. Training set MOR ligands for the agonist shape-based models and their binding affinity (as K_i) at the MOR (page S9).

Supplementary Chart S4. Training set MOR ligands for the antagonist shape-based models and their binding affinity (as K_i) at the MOR (page S10).

Supplementary Figure S1. Overview of the (a) pharmacophore- and (b) shape-based virtual screening workflow for MOR agonists and antagonists (page S11).

Supplementary Figure S2. Competitive inhibition of [^{3}H]DAMGO binding by selected virtual hits **T1-T15** and **1-3** at the human MOR (page S12).

Supplementary Table S1. Inactive virtual hits (page S13).

Supplementary Section S3. Results of bioactivity profiling (page S15).

Supplementary Section S4. Evaluation and discussion of the applied virtual screening methods (page S16).

Supplementary Figure S3. Comparison of Lys233 in the 4DKL and 5C1M structures (page S20).

Supplementary Figure S4. Comparison of the available crystal structures (page S21).

Supplementary Table S2. Structures of the agonists in the validation dataset (page S22).

Supplementary Table S3. Structures of the antagonists in the validation dataset (page S26).

Supplementary Table S4. Smiles codes of the "inactives" in the validation dataset (page S31).

Supplementary Section S5. Detailed description of the applied in silico tools (page S35).

References (page S41).

Supplementary Section S1. Detailed description of pharmacophore model generation.

Agonist modeling

The first agonist pharmacophore model was created with the reported crystal structure of the β-FNA-MOR complex.[1] It might seem unreasonable to generate an agonist model with an antagonist complex, however, this crystal structure may rather represent a semi-active state than an inactive one. The optimized model, pm-ag-4dkl-model-13 (Fig. 3a), mapped 15 out of 45 active compounds and no antagonists or inactive compounds. It had an enrichment factor (EF) of 5.3, which represents 100% of the maximum EF (maxEF). To be able to retain also the remaining active compounds and avoid the mapping of inactive compounds to the model, additional ligand-based models were generated.

The second pharmacophore model was built with the known MOR morphinane agonists **S1**[2] and **S2**[3] as well as the phenazocine derivative **S3**[4] (Supplementary Chart S1). The refined model pm-ag-lig-model-1 (Fig. 3b) matched 33 agonists, seven antagonists, and one inactive compound. Thereby, it retrieved an EF of 4.3, representing 81.0% of the maxEF.

The last agonist model was based on the oxymorphone analogues **S4**[2] and **S5**[5] (Supplementary Chart S1). After optimization, the model pm-ag-lig-model-2 (Fig. 3c), mapped 16 agonists, seven antagonists, and no inactive compound, leading to an EF of 3.7 (69.6% of the maxEF). This model is based on two morphinans (**S4** and **S5**) and since it contains three HBAs, it might represent a specific morphinan agonist model.

Antagonist modeling

In the course of the antagonist modeling, only a ligand-based approach was applied. The first two models were generated with the MOR antagonists naltrexone **2**[6], the piperidine derivative **S6**[7], and alvimopan **S7**[8] (Supplementary Chart S2), where in one model, the HBA had been exchanged for a HBD.

The first optimized ligand-based pm-ant-lig-model-3 (Fig. 3d) mapped 22 antagonists, 26 agonists, and no inactive compound. It retrieved an EF of 3.2 (62.7% of the maxEF). The second model based on these compounds, model pm-ant-lig-model-4 (Fig. 3e), matched 32 antagonists, 27 agonists, and 5 inactive compounds. This led to an EF of 3.5 or 68.6% of the maxEF. However, the two models matched also several agonists, therefore, they may rather be considered as general MOR ligand models than as selective MOR antagonist models.

The last MOR-antagonist model, pm-ant-lig-model-5, was based on the antagonists quinolizine derivative **S8**[9] and pyrazine derivative **S9**[10] (Supplementary Chart S2). The optimized model (Fig. 3f) mapped only 10 antagonists and no further compounds, thereby retrieving an EF of 5.1 (100% of the maxEF).

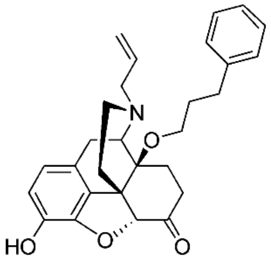

S1
K_i = 0.02 nM

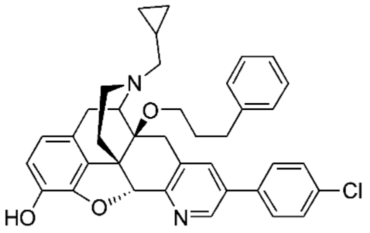

S2
K_i = 0.66 nM

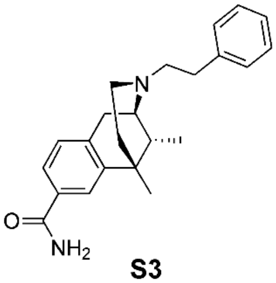

S3
K_i = 0.015 nM

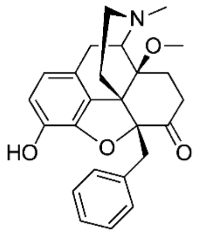

S4
K_i = 0.31 nM

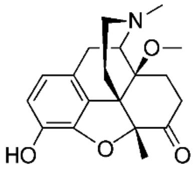

S5
K_i = 0.15 nM

Supplementary Chart S1. Training set MOR ligands for the agonist pharmacophore models and their binding affinities (as K_i) at the MOR.

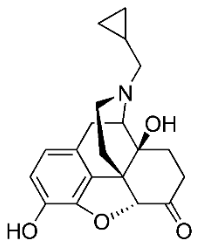

2

K_i = 0.20 nM

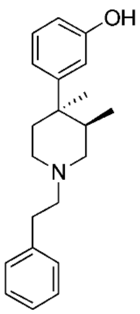

S6

K_i = 1.8 nM

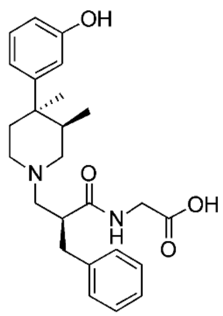

S7

K_i = 0.47 nM

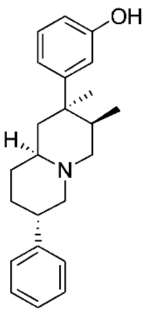

S8

K_i = 0.62 nM

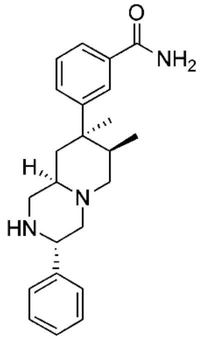

S9

K_i = 1.8 nM

Supplementary Chart S2. Training set MOR ligands for the antagonist pharmacophore models and their binding affinity (as K_i) at the MOR.

Supplementary Section S2. Detailed description of shape-based model generation.

Agonist modeling

The first shape-based agonist model was based on the MOR agonists 7-aminomorphan derivative **S10**[11] and phenazocine derivative **S3**[4] (Supplementary Chart S3). For that purpose, the two molecules had to be first aligned in LigandScout, since ROCS does not provide an alignment function. The aligned poses were then loaded into vROCS. After optimization, the emerged model, shape-ag-model-1 (Fig. 4a), mapped 12 of the 45 agonists, four antagonists, and no inactive compounds with a ComboScore of ≥ 1.02. This led to an EF of 4.0, representing 75.5% of the maxEF.

The second model was generated with one low-energy conformation of quinolizine derivative **S11**[9] (Supplementary Chart S3). The refined model shape-ag-model-2 (Fig. 4b) mapped 4 out of the 45 agonists and one further antagonist out of 47 with a cut-off ComboScore of ≥1.35, thereby retrieving an EF of 4.3 or 81.0% of the maxEF.

The last agonist model was created with a low-energy conformation of morphine **1**[12] (Supplementary Chart S3 and Fig. 4c). In this case, the default model was used, since further optimization steps did not lead to any improvements in the theoretical validation. The morphine model, named shape-ag-model-3, matched 19 agonists, 8 antagonists, and 3 inactive compounds with a ComboScore of ≥ 1.5. This led to an EF of 3.4 (63.3% of the maxEF).

Antagonist modeling

The first model was created with a low-energy conformation of the MOR antagonist alvimopan **S7** (Supplementary Chart S4 and Fig. 4d). After optimization, the final model, shape-ant-model-1 ranked 16 antagonists (34.0%), but only 3 out of 45 agonists (6.7%) and one out of 148 inactive compounds (0.7%), with a ComboScore of ≥ 1.35, leading to an EF of 4.1 (80.1% of the maxEF).

The second model (shape-ant-model-2) was generated with one low-energy conformation of the antagonist MCL-702 **S12**[13] (Supplementary Chart S4 and Fig. 4e). Similar to the morphine-based model, also the MCL-702 model could not be further improved and was applied as the default model. It mapped six antagonists and only one agonist above the cut-off value of 1.20, and retrieved an EF of 4.4 (85.8% of the maxEF).

The antagonist carboxamido biaryl ether derivative **S13**[14] (Supplementary Chart S4) served as query molecule for the generation of the next ROCS antagonist model. After refinement, it mapped three antagonists from the dataset, but no further compound above a ComboScore of 1.65. This model, named shape-ant-model-3 (Fig. 4f), retrieved the maxEF of 5.1.

The last model was generated with the aligned poses of naltrexone derivatives ALKS-33 **S14**[15] and **S15**[16] (Supplementary Chart S4 and Fig. 4g). All color features in close proximity were merged to one. The final model shape-ant-model-4 mapped 15 antagonists, and 7 agonists with a ComboScore of ≥ 1.20, leading to an EF of 3.5 (68.3% of the maxEF).

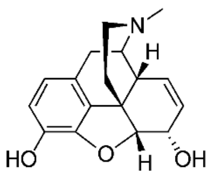

1
K_i = 4.9 nM

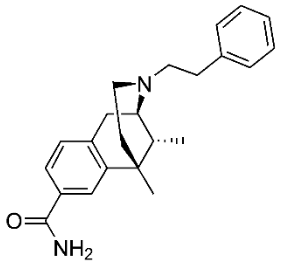

S3
K_i = 0.015 nM

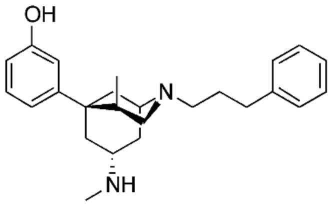

S10
K_i = 0.371 nM

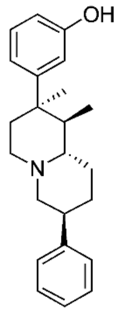

S11
K_i = 0.9 nM

Supplementary Chart S3. Training set MOR ligands for the agonist shape-based models and their binding affinity (as K_i) at the MOR.

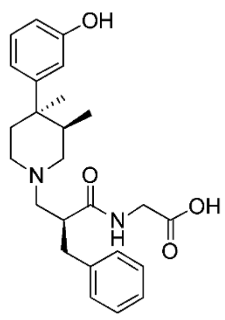

S7

K_i = 0.47 nM

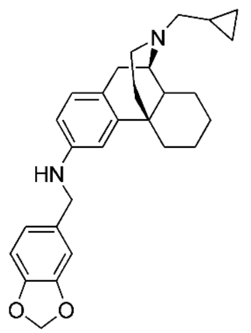

S12

K_i = 0.17 nM

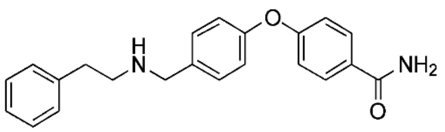

S13

K_i = 0.29 nM

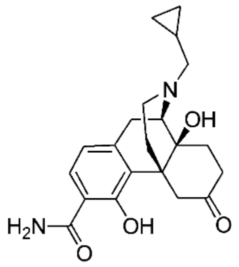

S14

K_i = 0.052 nM

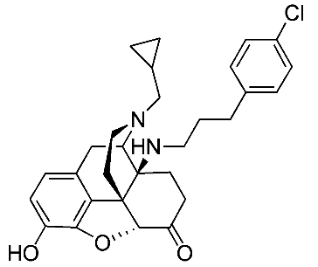

S15

K_i = 0.13 nM

Supplementary Chart S4. Training set MOR ligands for the antagonist shape-based models and their binding affinity (as K_i) at the MOR.

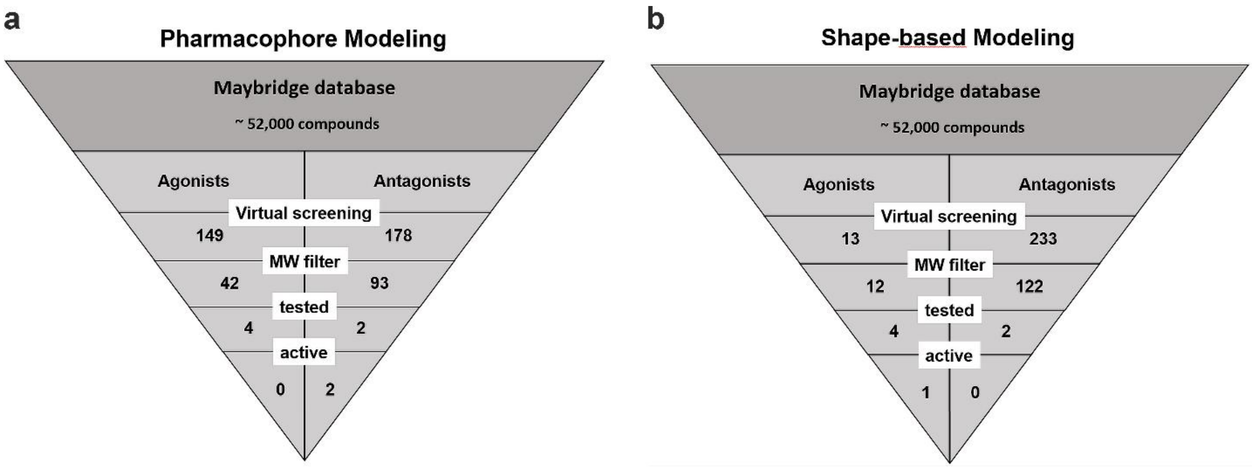

Supplementary Figure S1. Overview of the (a) pharmacophore- and (b) shape-based virtual screening workflow for MOR agonists and antagonists.

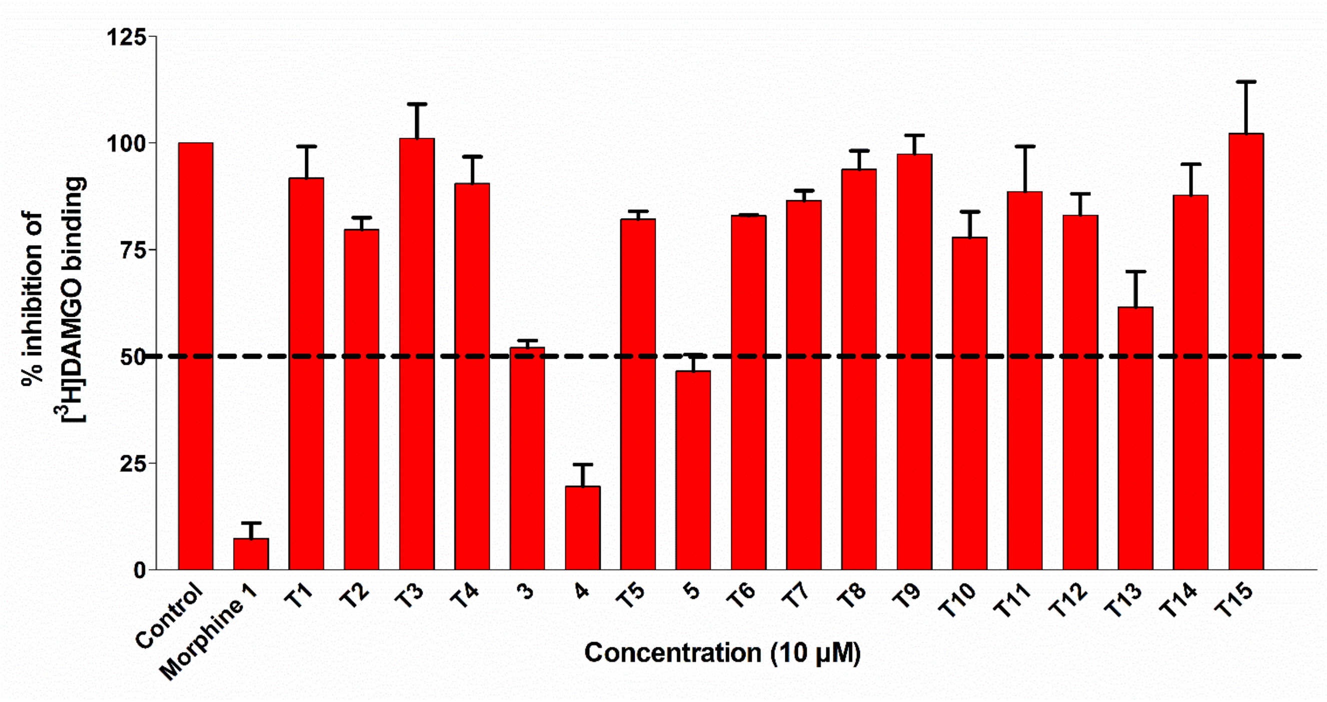

Supplementary Figure S2. **Competitive inhibition of [^{3}H]DAMGO binding by selected virtual hits T1-T15 and 3-5 at the human MOR.** Membranes of CHO cells stable transfected with the human MOR were incubated with [^{3}H]DAMGO (1 nM) at 25°C for 60 min in the absence (control) or presence of 10 µM of compounds **T1-T15, 3-5,** or morphine **1**. Three molecules **3**, **4** and **5** inhibited by ≥50% [^{3}H]DAMGO binding to CHO-hMOR cells. Experimental data were analyzed and graphically processed using the GraphPad Prism Software. All values are expressed as the mean ± SEM (n = 3). Nonvisible SEM is within the symbol.

Supplementary Table S1. Inactive virtual hits.

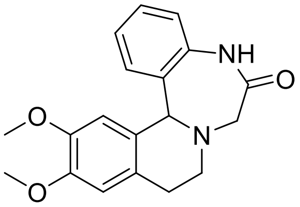	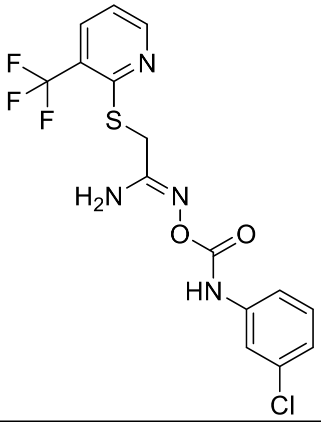	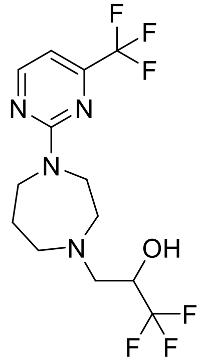
Compound **T1**	Compound **T2**	Compound **T3**
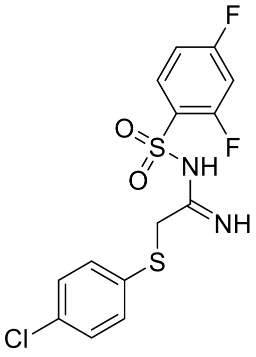	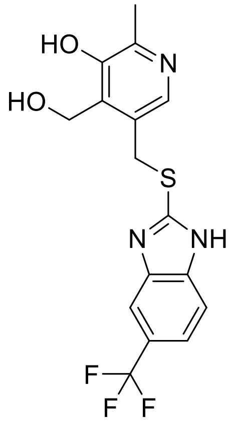	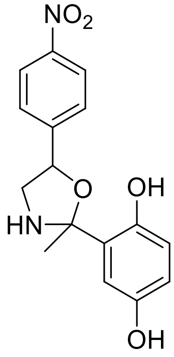
Compound **T4**	Compound **T5**	Compound **T6**
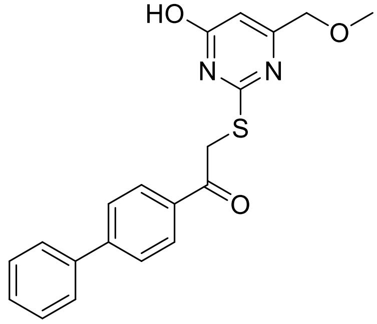	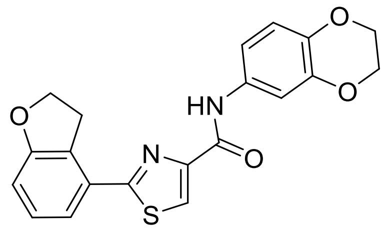	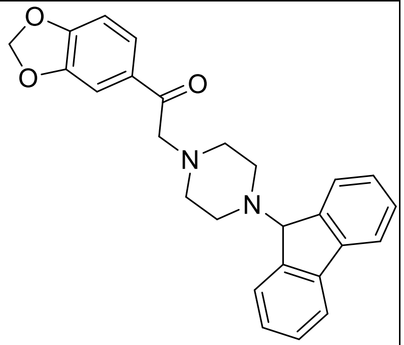
Compound **T7**	Compound **T8**	Compound **T9**

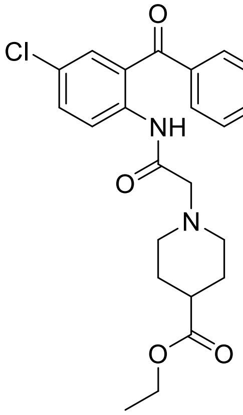	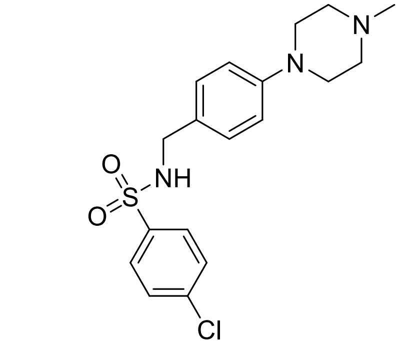	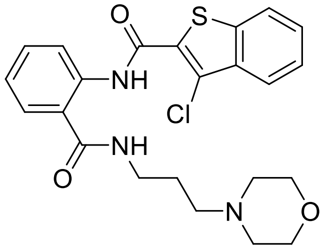
Compound **T10**	Compound **T11**	Compound **T12**
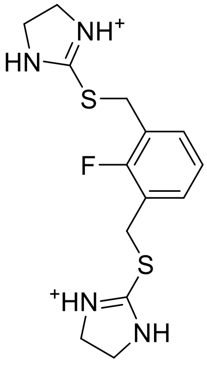	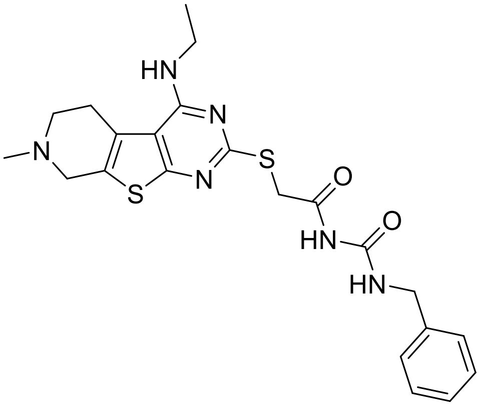	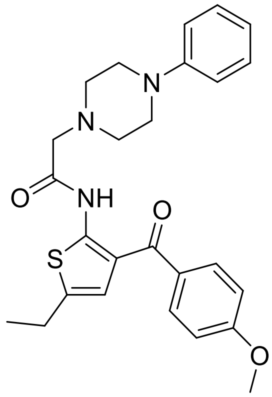
Compound **T13**	Compound **T14**	Compound **T15**

Supplementary Section S3. Results of bioactivity profiling.

All 18 compounds were profiled with the 2D-similarity-based programs SEA[17] and PASS,[18] and the external pharmacophore-based profiling tool PharmMapper[19] to investigate whether these compounds were also predicted to display MOR activity by these applied tools. The PharmaDB[20] did not contain models for the MOR, therefore no predictions could be generated. However, none of the compounds was predicted by at least one of other the profiling tools above the required activity cut-off.

Supplementary Section S4. Evaluation and discussion of the applied virtual screening methods.

As results of different computational approaches, two active ligands at the MOR, **3** and **4**, were identified with pharmacophore modeling one molecule, **5**, was identified with shape-based modeling, and no active compound was among the top-ranked docking hits. It is well-known that docking is often limited by scoring and the correct ranking of compounds.[21] Since we selected our test compounds exclusively based on the GoldScore and the predicted interaction patterns, and did not include any manual inspection to avoid bias, this outcome was not surprising. In our recent study on cyclooxygenases (COX),[22] also docking performed modestly compared to the other methods when it came to enrichment of active compounds at the top of the hitlist. However, we identified one active molecule with a totally distinct chemical structure from all so far known COX-inhibitors.[22] This compound was ranked within the top-20 at position 16, so since we could only test the top-two and top-four ranked compounds, respectively, we might have missed actually active compounds ranked lower. For projects that aim at identifying structurally novel compounds, and therefore, a higher number of false positive hits can be accepted, docking might still offer advantages.

Although docking did not directly contribute to the identification of novel MOR ligands, still, the docking-based investigations represented the crucial pre-requisite for the generation of the pharmacophore- and shape-based models. The detailed analysis of the docking poses of agonists, antagonists, and inactive compounds, allowed for further insights into the mode of action, and without that knowledge, the previous model generation had failed.

The agonist pharmacophore model generation performed very well, especially when a lot of XVols were added. However, compounds that are not allowed to protrude into a certain area of the MOR binding site also cannot form interactions in this site, which might explain the importance of the XVols for the agonist modeling. Despite the good performance of the models in the theoretical validation, they did not

retrieve any active molecules in the prospective part of the study. Again, this might be attributed to the low amount of hits selected for biological testing.

The MOR antagonist pharmacophore modeling appeared to be much more complex, as two of the models could not discriminate between agonists and antagonists in the theoretical validation. These two models may therefore rather represent general MOR ligand models than specific antagonist models. Those two models also predicted the two top-ranked hits selected for biological testing. In detail, **3** mapped model pm-ant-lig-model-3, while **4** matched model pm-ant-lig-model-4. Interestingly, both molecules were found to be active at the MOR in the biological testing. The performance of the antagonist models is especially notable, considering the limited amount of experimental data.

Both agonist and antagonist shape-based modeling strategies proved to be very effective in the theoretical validation, and in addition the antagonist shape-models could discriminate between agonists and antagonists. This can be explained by the different definition of a "hit" applied in ROCS[23,24] and LigandScout.[25] In LigandScout, a compound needs to map all feature included in the model to be considered as hit. Subsequently, a pharmacophore fit value is assigned, that determines how well the compound maps the model. In ROCS, a score is assigned for every compound of the screening database, and the number of the final output results is user-defined. However, that implies that a compound does not have to match all features included in the model to be ranked. According to our hypothesis, there might be multiple options for antagonists to form additional polar interactions and prevent the activation of the receptor. It might be difficult to cover all these potential interactions, and the combinations thereof, in a few pharmacophore models as every compound needs to fulfill the whole hypothesis to be considered as hit. However, every compound that matches an additional polar color feature in a ROCS model (potential antagonist) gets ranked at a higher position as compounds that do not (potential agonists). Therefore, ROCS models may have advantages especially for projects, where it is not clear which polar interactions might actually be crucial for binding of a ligand and which ones can be neglected, or for

projects, where many very similar binding modes can occur. In addition, also the activity of compound **5** predicted with the ROCS agonist model shape-ag-model-2 could be confirmed in the biological assessments. However, this compound displayed antagonistic activity.

To further examine the hit lists derived from the different applied methods we analyzed whether top-ranked compounds from one method were also predicted by other methods above the activity cut-off. Several of these compounds were consensus hits, however, none of them was active in the biological evaluation. Specifically, **T13** (predicted as agonist by docking) also mapped an antagonist pharmacophore model, the ROCS agonist hit **T6** also mapped an agonist pharmacophore model and was predicted as agonist by docking with a GoldScore of 52.8, the agonist pharmacophore modeling hit **T4** also fulfilled the docking criteria for agonists and retrieved a GoldScore of 44.4, and the agonist pharmacophore hit **T2** was predicted as antagonist in docking since it formed additional interactions with Gln124 at a GoldScore of 57.9. This finding is in contrast to our previous study on COX,[22] where we could observe a strong correlation relationship between the number of methods that predicted a compound to be active and the actual activity in the experimental setting. Nevertheless, also in the same study[22] we have observed some cases where methods contributed to the identification of novel active molecules alone, and such findings were also reported by others.[26]

The structural diversity compared to the known active ligands at the MOR may also explain the limited performance observed for the applied bioactivity profiling tools. The investigation of different ChEMBL[27] datasets in the course of the CYP study[28] highlighted the dependence of the 2D similarity-based tool SEA[17] on the underlying dataset of known active compounds.[28] An increasing number of active compounds was correctly predicted with increasing version of the ChEMBL database. Also, the experimental data available for the prediction increased in higher ChEMBL versions, thereby suggesting that the performance of the tool can be improved with the addition of further biological activity data.

Since the ligands identified in the present work represent novel scaffolds, no active compounds might be included in the dataset that cover also these structural classes.

In the case of PharmMapper,[19] where also no molecule was mapped to a MOR pharmacophore model, we assume that this target may not be included in the pharmacophore model collection since the crystal structure was published not until recently.[1] To further evaluate this aspect, we profiled the co-crystallized MOR ligand β-FNA, which should under any circumstances map the model. However, also β-FNA was not predicted to interact with the MOR. Since the underlying reason remains speculative, we highly encourage the distributors of bioactivity profiling tools to provide information about the targets that are represented by their software.

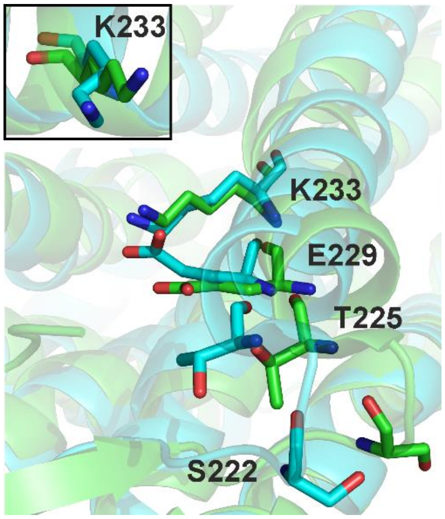

Supplementary Figure S3. Comparison of Lys233 in the 4DKL[1] and 5C1M[29] structures. Lys233 in 5C1M is free to move, and although only a small shift can be observed (top corner), this subsequently leads to pronounced conformational adaptions of neighboring residues, which are connected via a polar interaction network.

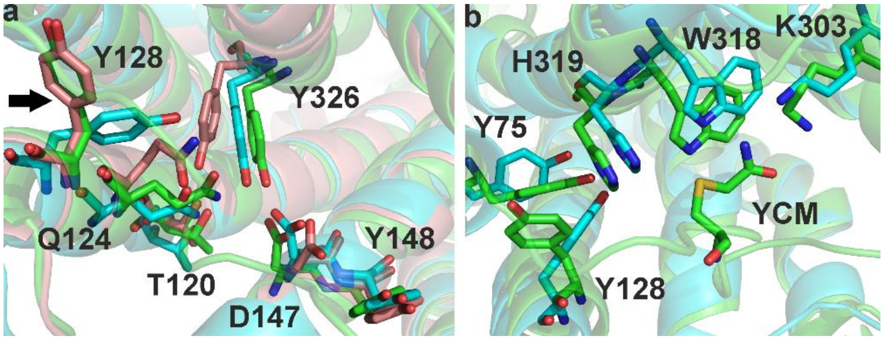

Supplementary Figure S4. Comparison of the available crystal structures. (a) The comparison of the binding pockets of 4DKL[1] (MOR in complex with β-FNA, cyan), 4DJH[30] (KOR, salmon), and 5C1M[29] (MOR in complex with BU72, green), revealed almost identical binding sites of the two MOR structures, whereas the KOR structure differs. The only exception is Tyr128 as indicated by the black arrow, which adopts the conformation of the KOR. This is caused by YCM (b), an artificial disulfide bond in the N-terminus absent in the 4DKL structure (cyan), which interacts with K303 in 5C1M (green). This novel interaction disrupts the cation-pi interaction between K303 and W318 in the 4DKL structure, leading to a shift of W318 and H319 in 5C1M. AS a consequence, the hydrogen bond between H319 and Y128 in 4DKL is disrupted as well and Y75 adapts its conformation to form a hydrogen bond with H319. Y128 in the 4DKL structure would clash with both Y75 and H319 in 5C1M. Due to this steric hindrance, Y128 needs to fold towards the KOR conformation. W318 and H319 were not included in the comparison in Fig. 2C, because they are not conserved in the KOR.

Supplementary Table S2. Test set compounds for the MOR agonist modeling.

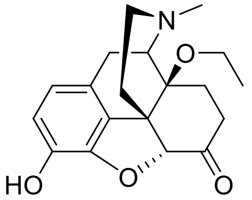	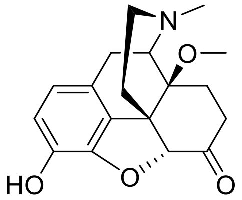	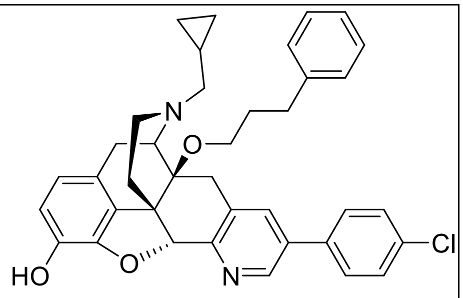
14-*O*-ethyloxymorphone[2]	14-*O*-methyloxymorphone[31]	14-alkoxypyridomorphinan derivative **S2**[3]
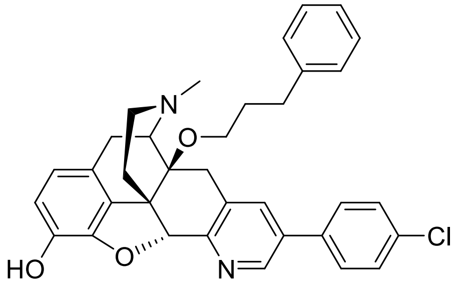	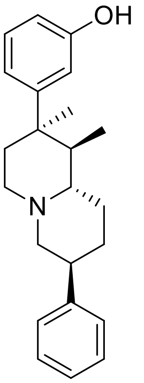	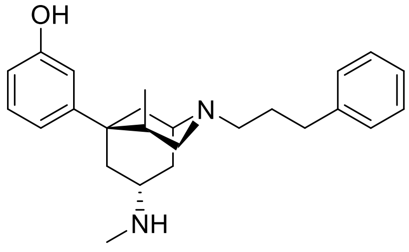
14-alkoxypyridomorphinan derivative[3]	octahydroquinolizine derivative **S11**[9]	7-aminomorphan derivative **S10**[11]
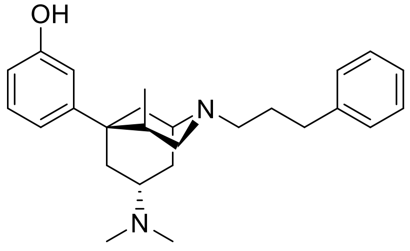	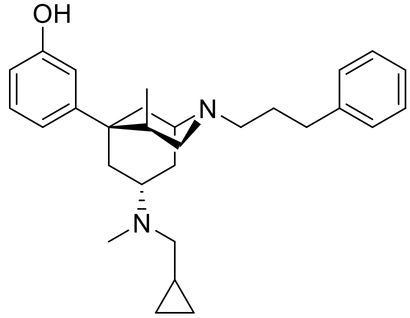	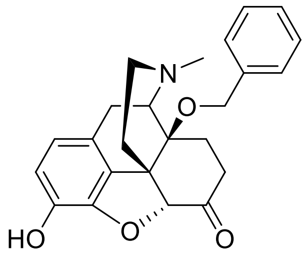
7-aminomorphan derivative[11]	7-aminomorphan derivative[11]	4-*O*-arylalkyloxymorphone derivative[2]
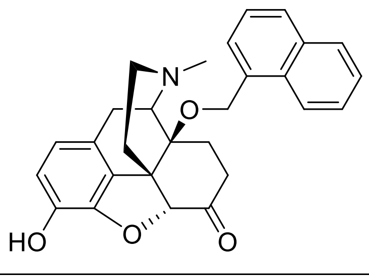	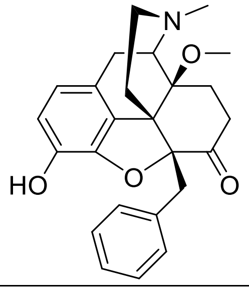	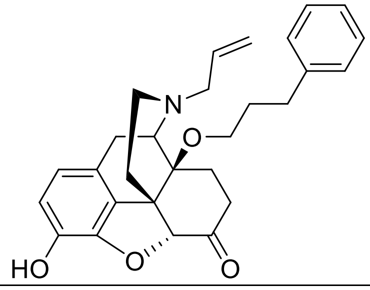
4-*O*-arylalkyloxymorphone derivative[2]	14-alkoxy-*N*-methylmorphinan-6-on derivative **S4**[2]	14-phenylpropoxymorphinan-6-on derivative **S1**[2]

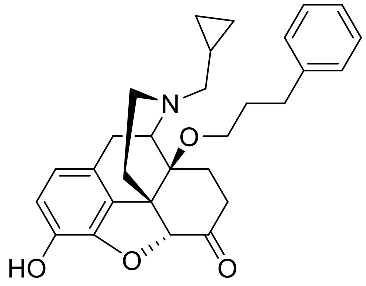	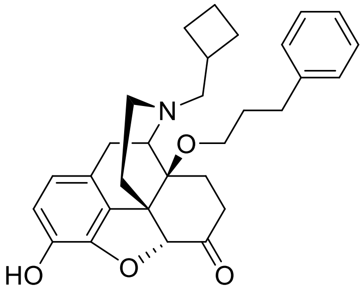	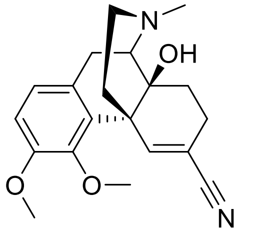
morphinan-6-one derivative[2]	morphinan-6-one derivative[2]	didehydromorphinan-6-carbonitrile derivative[2]
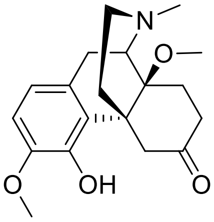	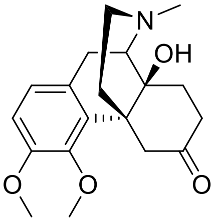	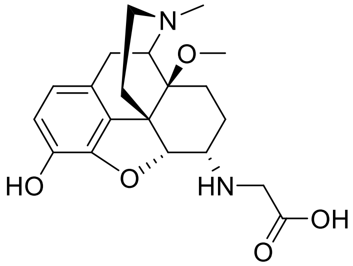
6-keto-morphinan[2]	6-keto-morphinan[2]	14-methoxymorphinan derivative[2]
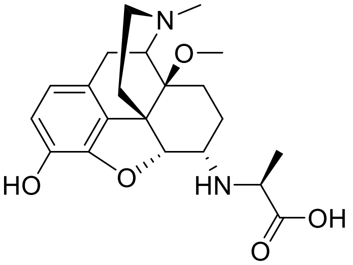	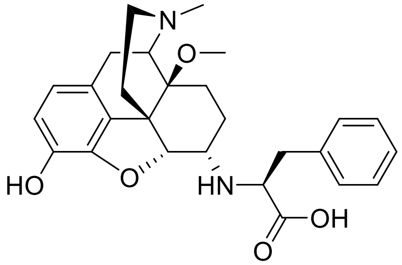	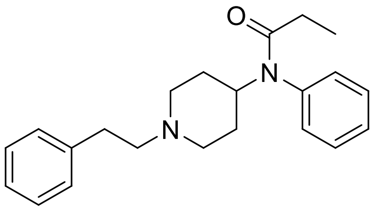
14-methoxymorphinan derivative[2]	14-methoxymorphinan derivative[2]	fentanyl[32]
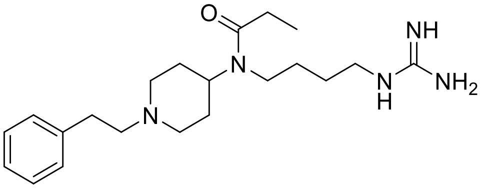	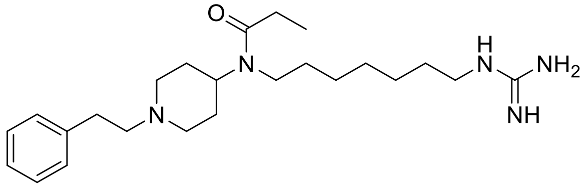	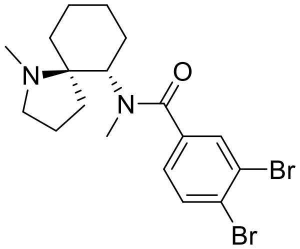
piperidine derivative[32]	piperidine derivative[32]	1-azaspiro[4.5]decan-10-yl-amide[33]
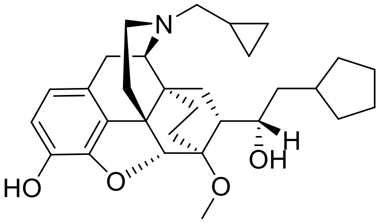	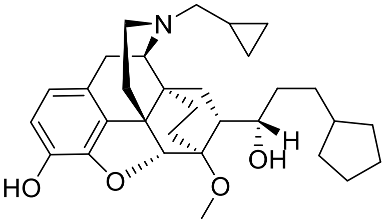	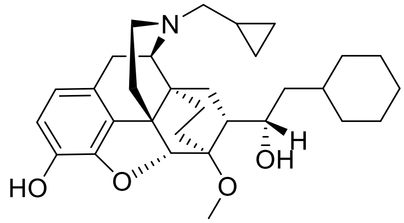
6,14-ethenmorphinan derivative[34]	6,14-ethenmorphinan derivative[34]	6,14-ethenmorphinan derivative[34]

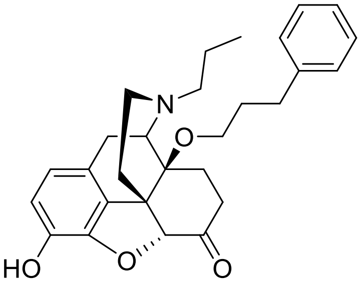	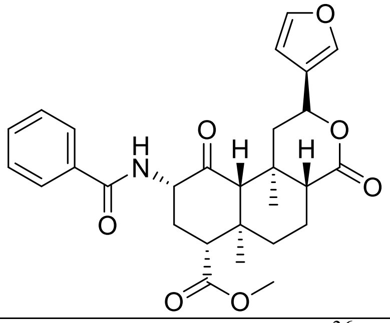	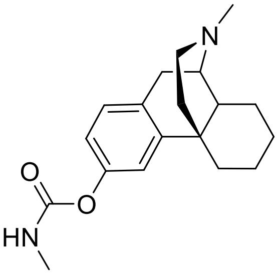
morphinan-6-one derivative[35]	salvinorin A derivative[36]	MCL-433[37]
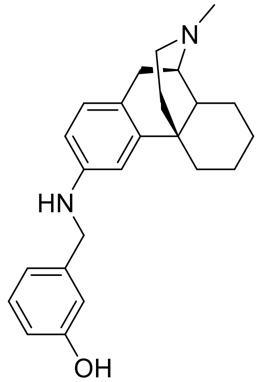	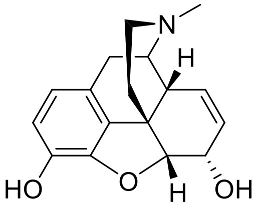	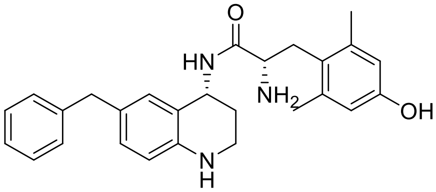
MCL-725[13]	morphine **1**[12]	tetrahydroquinolin derivative[38]
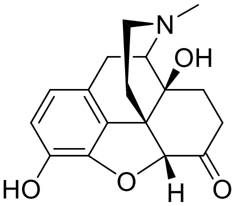	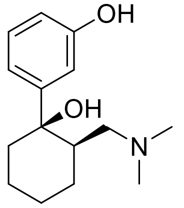	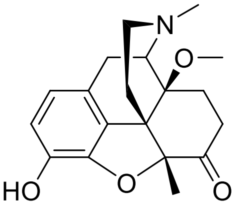
oxymorphone[5]	active tramadol metabolite[39]	oxymorphone derivative **S5**[5]
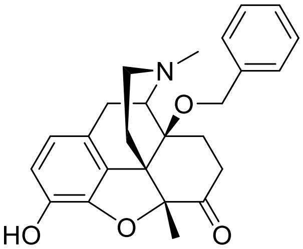	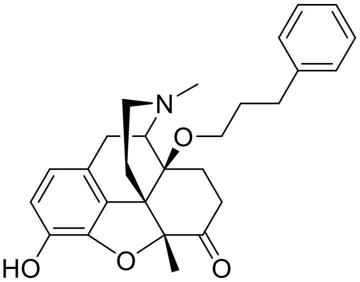	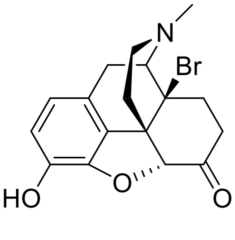
oxymorphone derivative[5]	oxymorphone derivative[5]	14β-bromodihydromorphinone[40]
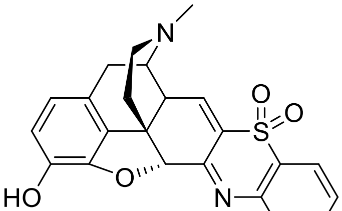	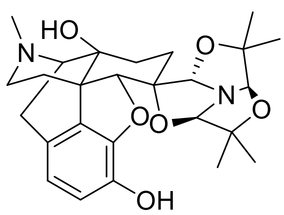	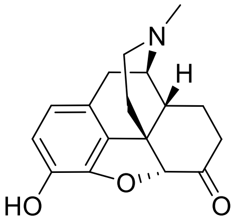
benzothiazinomorphinan[40]	SYK-385[41]	hydromorphone derivative[15]

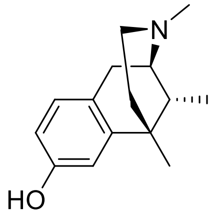	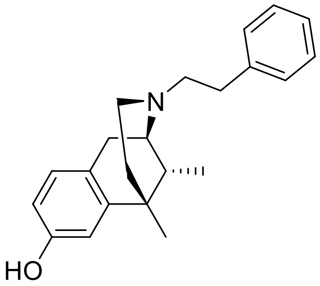	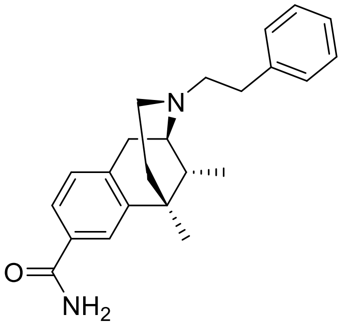
metazocine derivative[4]	phenazocine derivative[4]	phenazocine derivative **S3**[4]

Supplementary Table S3. Test set compounds for the MOR antagonist modeling.

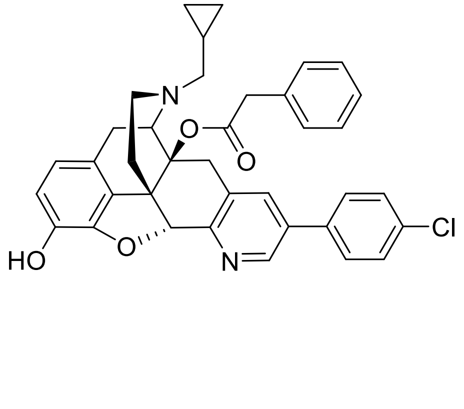	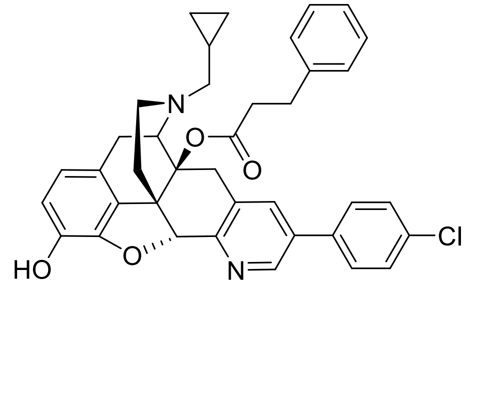	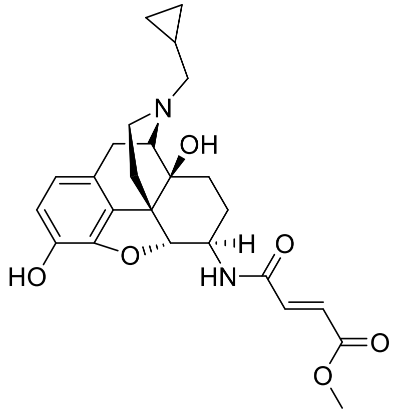
14-Acyloxypyridomorphinan derivative[3]	14-Acyloxypyridomorphinan derivative[3]	β-funaltrexamine[1]
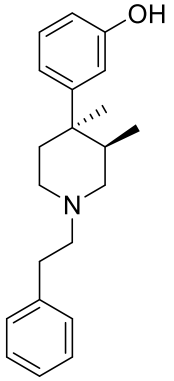	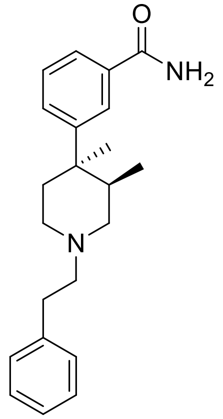	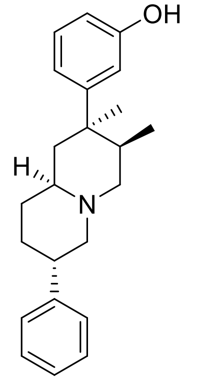
piperidine derivative **S6**[7]	piperidine derivative[7]	octahydroquinolizine derivative **S8**[9]
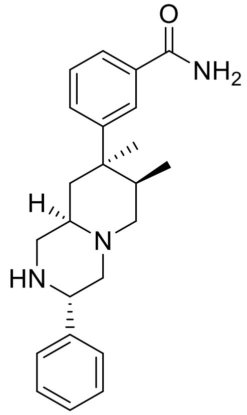	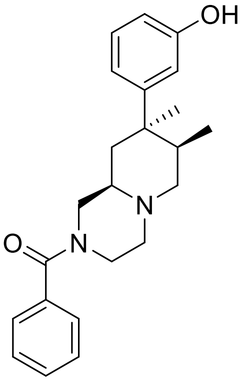	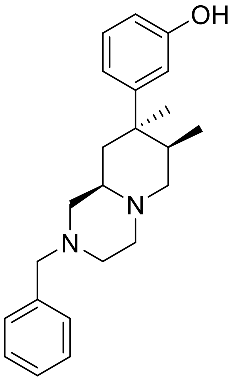
pyrazine derivative **S9**[10]	pyrazine derivative[10]	pyrazine derivative[10]

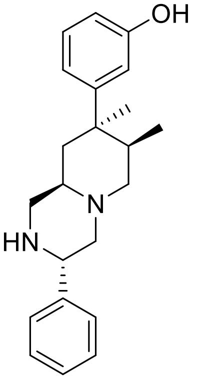	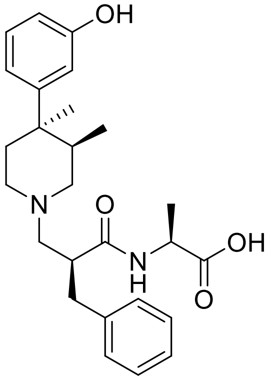	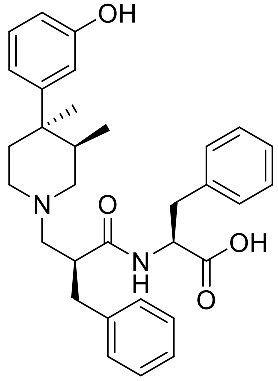
pyrazine derivative[10]	piperidine derivative[8]	piperidine derivative[8]
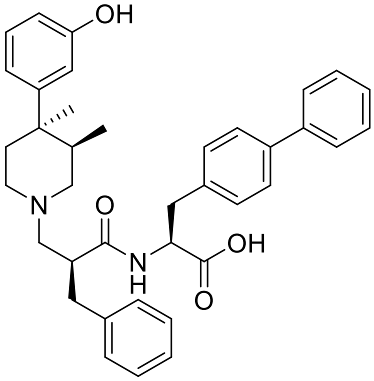	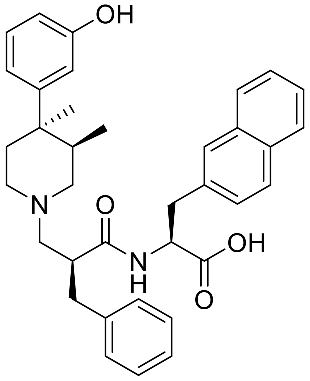	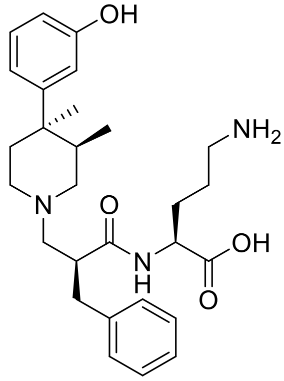
piperidine derivative[8]	piperidine derivative[8]	piperidine derivative[8]
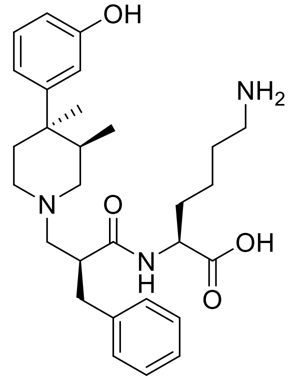	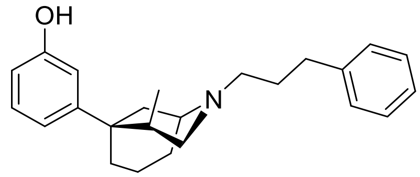	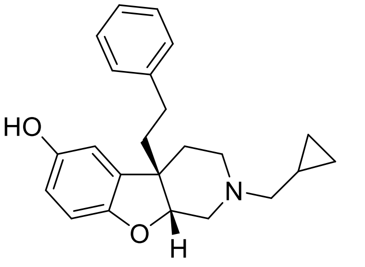
piperidine derivative[8]	N-phenylpropyl-4β-methyl-5-(3-hydroxyphenyl)morphan[11]	hexahydrobenzofuro[2,3-c]pyridin-6-ol derivative[42]
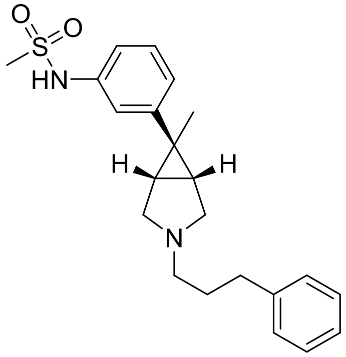	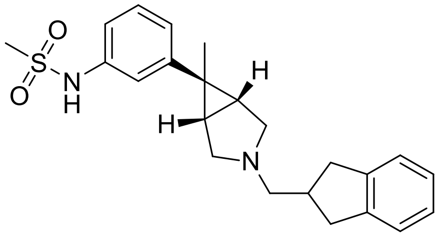	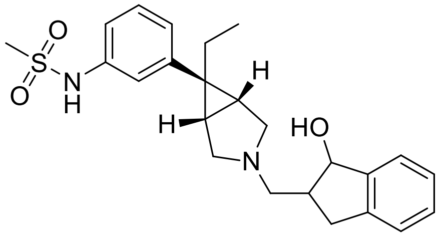
3-azabicyclo[3.1.0]hexane derivative[43]	3-azabicyclo[3.1.0]hexane derivative[43]	3-azabicyclo[3.1.0]hexane derivative[43]

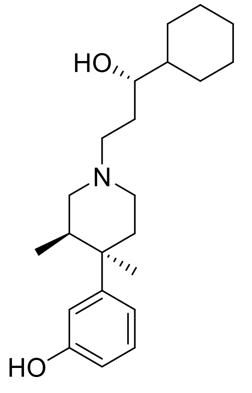	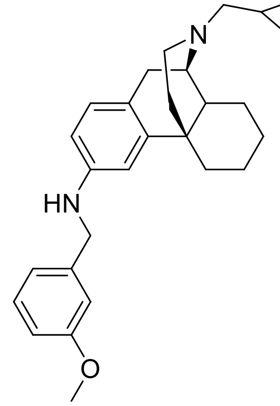	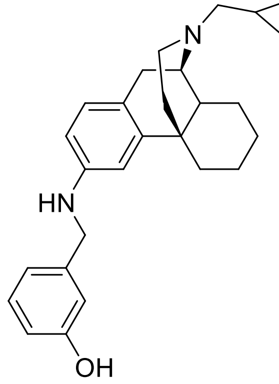
LY255582[14]	MCL-700[13]	MCL-701[13]
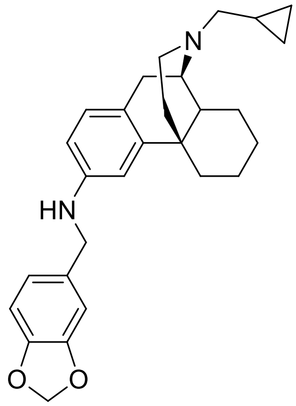	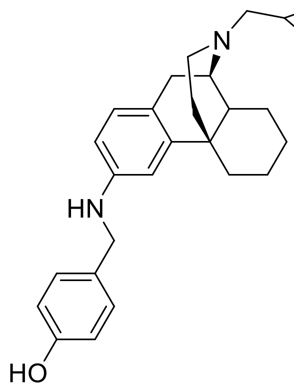	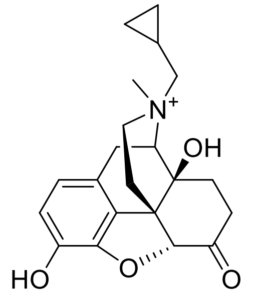
MCL-702 **S12**[13]	MCL-703[13]	methylnaltrexone[15]
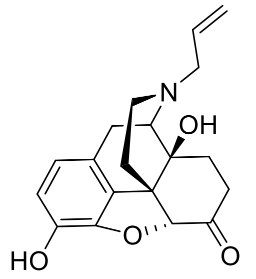	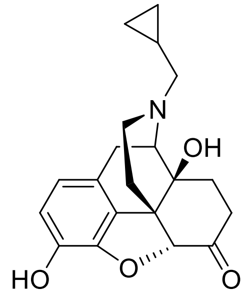	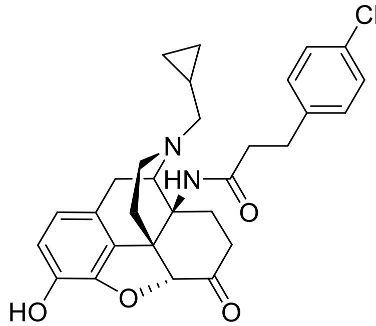
naloxone[9]	naltrexone **2**[6]	14-aminodihydromorphinone derivative[16]
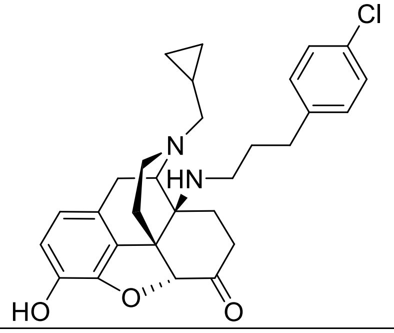	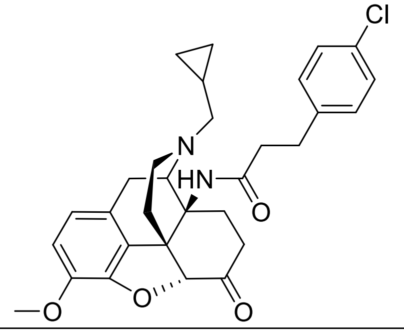	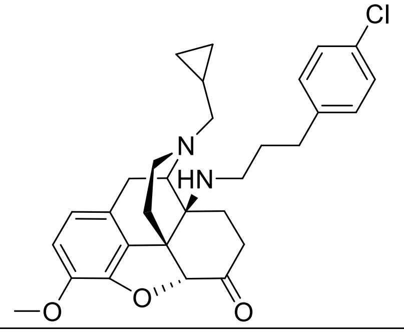
14-aminodihydromorphinone derivative **S15**[16]	14-aminodihydromorphinone derivative[16]	14-aminodihydromorphinone derivative[16]

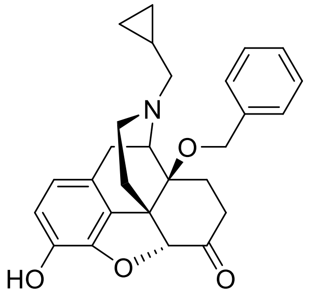	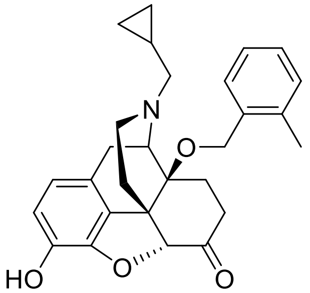	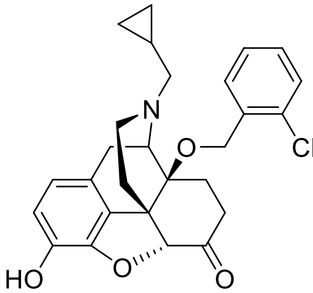
14-*O*-benzylnaltrexone[6]	14-*O*-benzylnaltrexone derivative[6]	14-*O*-benzylnaltrexone derivative[6]
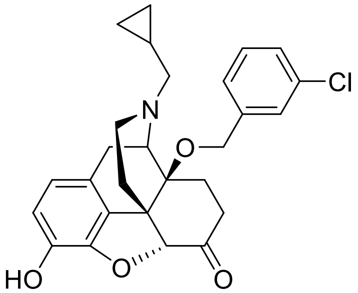	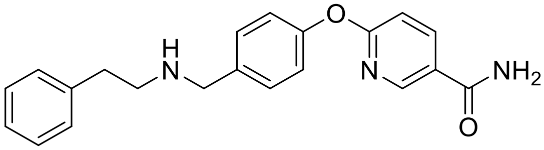	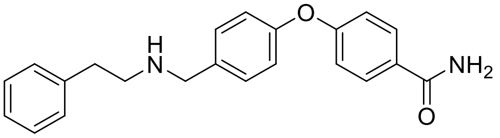
14-*O*-benzylnaltrexone derivative[6]	carboxamido-biaryl ether derivative[14]	carboxamido-biaryl ether derivative **S13**[14]
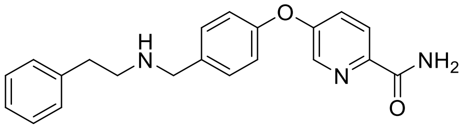	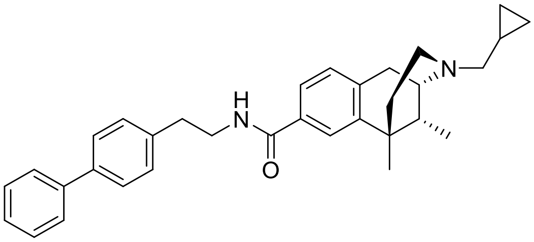	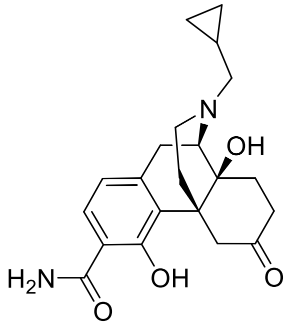
carboxamido-biaryl ether derivative[14]	N-BPE-8-CAC[44]	ALKS-33 **S14**[15]
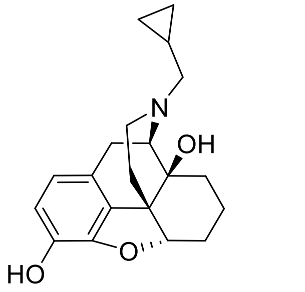	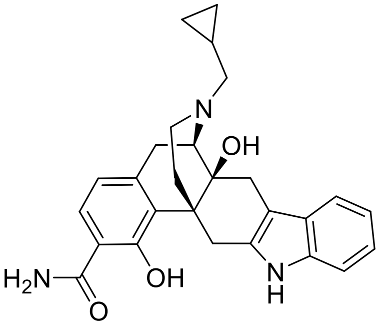	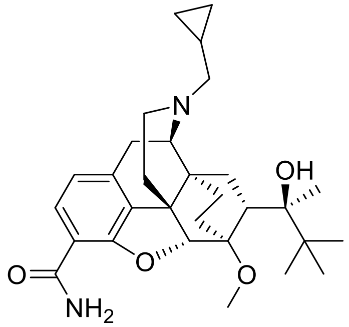
6-desoxonaltrexone derivative[15]	naltrindole derivative[15]	buprenorphine derivative[4]

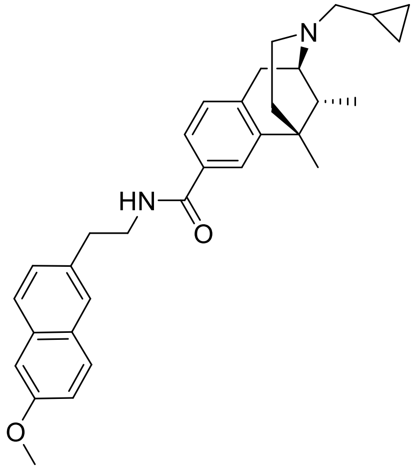	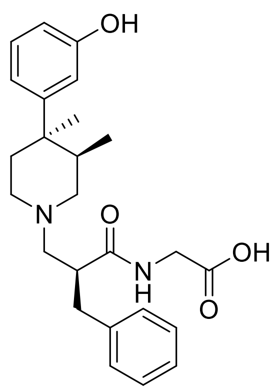	
2,6-methano-3-benzazocine derivative[45]	alvimopan **S7**[8]	

Supplementary Table S4. Smiles codes of the molecules in the "inactives" dataset.

c1ccc(cc1)CCN1CC[C@@](c2cccc(c2)C(=O)N(C)C)([C@H](C1)C)C
c1ccc(cc1)CN1CCCN([C@@H](CO)C1)Cc1ccccc1
c1ccc(cc1)CN1CCCN(C[C@@H]1CO)CC(OC)OC
c1cc(cc(c1)OC)[C@H](N1C[C@H](N(C[C@@H]1C)CC=C)C)c1ccc(cc1)C(=O)N(CC)CC
c1cc(cc(c1)F)[C@H](N1C[C@H](N(C[C@@H]1C)CC=C)C)c1ccc(cc1)C(=O)N(CC)CC
c1cc(cc(c1)I)[C@H](N1C[C@H](N(C[C@@H]1C)CC=C)C)c1ccc(cc1)C(=O)N(CC)CC
c1ccc(cc1)[C@@H](N1C[C@H](N(C[C@@H]1C)CC=C)C)c1ccc(cc1)C(=O)N(CC)CC
c1cc(cc(c1)OC)[C@H](N1C[C@H](N(C[C@@H]1C)CC=C)C)c1ccc(cc1)C(=O)N(C)C
c1cc(cc(c1)OC)[C@H](N1C[C@H](N(C[C@H]1C)CC=C)C)c1ccc(cc1)C(=O)N(CC)CC
c1cc(cc(c1)OC)[C@H](N1C[C@H](N(C[C@@H]1C)CC=C)C)c1ccc(cc1)C(=O)N(C)CC
c1ccc(cc1)C(=C1CCNCC1)c1ccc(cc1)C(=O)N(CC)CC
c1cc(cc(c1)OC)C(=C1CCNCC1)c1ccc(cc1)C(=O)N(CC)CC
c1ccc(cc1)C(=C1CCN(CC1)CC=C)c1ccc(cc1)C(=O)N(CC)CC
c1cc(cc(c1)F)C(=C1CCNCC1)c1ccc(cc1)C(=O)N(CC)CC
c1ccc(cc1)C(=C1CCN(CC1)CC=C(C)C)c1ccc(cc1)C(=O)N(CC)CC
c1cc(ccc1CC(=O)N([C@@H]1[C@@H](N(C)C)CCc2cc(c(cc12)OC)OC)CCOC)Cl
c1ccc(cc1)CCN1CC[C@@H](CC1)N(c1cccc(c1)NC1=NCCN1)C(=O)CC
c1ccc(cc1)CCN1CC[C@@H](CC1)N(C(=O)CC)CCCNC1=NCCN1
c1ccc(c(c1)C(=O)N[C@@H]1[C@@]2(N(CCC2)C)CCCC1)Br
c1cc(ccc1C(=O)N[C@@H]1[C@@]2(N(CCC2)C)CCCC1)F
c1cc(cc(c1)Br)C(=O)N[C@@H]1[C@@]2(N(CCC2)C)CCCC1
c1ccc(cc1)CCC(=O)N[C@@H]1[C@@]2(N(CCC2)C)CCCC1
c1cc(ccc1C(=O)N[C@@H]1[C@@]2(N(CCC2)C)CCCC1)OC
c1cc(ccc1S(=O)(=O)N[C@@H]1[C@@]2(N(CCC2)C)CCCC1)Br
c1ccc(cc1)CCN1CC[C@@H](CC1)N(C(=O)CC)CCCNC(=O)c1ccc2c(ccc(C3=NCCN3)n2)c1
c1ccc(cc1)CCN1CC[C@@H](CC1)N(C(=O)CC)CCCCCCNC(=O)c1ccc2c(ccc(C3=NCCN3)n2)c1
c1ccc(c(c1)C(=O)Nc1ccc2c(c1)c(cc(C)n2)N)COc1ccc(cc1)CNCCC
c1ccc(c(c1)C(=O)Nc1ccc2c(c1)c(cc(C)n2)N)COc1ccc(cc1)CNCCCC
c1ccc(c(c1)C(=O)Nc1ccc2c(c1)c(cc(C)n2)N)COc1ccc(cc1)CNCCCCC
c1ccc(c(c1)C(=O)Nc1ccc2c(c1)c(cc(C)n2)N)COc1ccc(cc1)CNCCCCCC
c1ccc(c(c1)C(=O)Nc1ccc2c(c1)c(cc(C)n2)N)COc1ccc(cc1)CNCCCCCCC
c1ccc(c(c1)C(=O)Nc1ccc2c(c1)c(cc(C)n2)N)COc1ccc(cc1)CNCCCCCCCC
c1ccc(c(c1)C(=O)Nc1ccc2c(c1)c(cc(C)n2)N)COc1ccc(cc1)CNCCCCCCCCC
c1ccc(c(c1)C(=O)Nc1ccc2c(c1)c(cc(C)n2)N)COc1ccc(cc1)CNCCCCCCCCCC
c1ccc(c(c1)C(=O)Nc1ccc2c(c1)c(cc(C)n2)N)COc1ccc(cc1)CNCCCN
c1ccc(c(c1)C(=O)Nc1ccc2c(c1)c(cc(C)n2)N)COc1ccc(cc1)CNCCCCN
c1ccc(c(c1)C(=O)Nc1ccc2c(c1)c(cc(C)n2)N)COc1ccc(cc1)CNCCCCCN
c1ccc(c(c1)C(=O)Nc1ccc2c(c1)c(cc(C)n2)N)COc1ccc(cc1)CNCCCCCCN
c1ccc(c(c1)C(=O)Nc1ccc2c(c1)c(cc(C)n2)N)COc1ccc(cc1)CNCCCCCCCN
c1ccc(c(c1)C(=O)Nc1ccc2c(c1)c(cc(C)n2)N)COc1ccc(cc1)CC
c1ccc(c(c1)C(=O)Nc1ccc2c(c1)c(cc(C)n2)N)COc1ccc(cc1)CNCCCCCCCCCN
c1cc(cc(c1)OC)[C@H](N1C[C@H](N(C[C@@H]1C)CC=C)C)c1ccc(cc1)C(=O)N(C(C)C)C(C)C
c1cc(cc(c1)OC)[C@H](N1C[C@H](N(C[C@@H]1C)CC=C)C)c1ccc(cc1)C(=O)N(CCC)CCC

c1ccc(c(c1)C(=O)Nc1ccc2c(c1)c(cc(C)n2)N)COc1ccc(cc1)CNCCCCCCCCCCN
c1cc(cc(c1)OC)[C@H](N1C[C@H](N(C[C@@H]1C)CC=C)C)c1ccc(cc1)C(=O)N1CCCC1
c1cc(cc(c1)OC)[C@H](N1C[C@H](N(C[C@@H]1C)CC=C)C)c1ccc(cc1)C(=O)N(CC)CCCC
c1cc(cc(c1)OC)[C@H](N1C[C@H](N(C[C@@H]1C)CC=C)C)c1ccc(cc1)C(=O)N1CCCCC1
c1ccc(cc1)CN1CC[C@@H](CC1)n1c2ccccc2[nH]c1=O
c1ccc(cc1)CN1C(=S)SCN(C1)Cc1ccccc1
c1cc(cc(c1)OC)[C@H](N1C[C@H](N(C[C@@H]1C)CC=C)C)c1ccc(cc1)C(=O)N1CCCCCC1
c1c2c(cc(c1Cl)N1CCN(C[C@H]1C)CCO)[nH]c(n2)SC(C)(C)C
c1c2c(cc(c1Cl)N1CCN(CC1)CC)[nH]c(C(=O)[C@]1(CC[C@@](CC1)(C)O)C)n2
c1cocc1[C@H]1OC(=O)[C@H]2[C@@]([C@H]3C(=O)[C@@H](OC(=O)C)C[C@@H](C(=O)O
C)[C@@]3(CC2)C)(C1)C
C1(=O)[C@@H]2[C@@]3([C@H](C(=O)O[C@H]([C@H]4COCC4)C3)CC[C@]2([C@H](C(=O
)OC)C[C@@H]1OC(=O)C)C)C
c1cc(cc(c1)OC)[C@H](N1[C@@H]2[C@H]3N([C@@H]([C@H]1CC2)CC3)CC=C)c1ccc(cc1)C(
=O)N(CC)CC
c1cc(cc(c1)OC)[C@H](N1[C@@H]2[C@H]3N([C@@H]([C@H]1CC2)CC3)CC=C)c1ccc(cc1)C(
=O)N(C1CCCCC1)CC
c1cocc1[C@H]1OC(=O)[C@H]2[C@@]([C@H]3C(=O)[C@@H](OC(=O)CCC(=O)O)C[C@@H
](C(=O)OC)[C@@]3(CC2)C)(C1)C
C1(=O)[C@@H]2[C@@]3([C@H](C(=O)O[C@H](C(=O)N[C@H]4COCC4)C3)CC[C@]2([C@
H](C(=O)OC)C[C@@H]1OC(=O)C)C)C
c1cocc1[C@H]1OC(=O)[C@H]2[C@@]([C@H]3C(=O)[C@@H](OC(=O)/C=C\C(=O)O)C[C@
@H](C(=O)OC)[C@@]3(CC2)C)(C1)C
c1cocc1[C@H]1OC(=O)[C@H]2[C@@]([C@H]3C(=O)[C@@H](OC(=O)CCCCC(=O)OC)C[C
@@H](C(=O)OC)[C@@]3(CC2)C)(C1)C
c1cocc1[C@H]1OC(=O)[C@H]2[C@@]([C@H]3C(=O)[C@@H](OC(=O)CCCC(=O)O)C[C@@
H](C(=O)OC)[C@@]3(CC2)C)(C1)C
C(#N)SCC(=O)O[C@@H]1C(=O)[C@@H]2[C@@]3([C@H](C(=O)O[C@H](c4ccoc4)C3)CC[C
@]2([C@H](C(=O)OC)C1)C)C
c1cocc1[C@H]1OC(=O)[C@H]2[C@@]([C@H]3C(=O)[C@@H](OC(=O)CC(=O)OC)C[C@@H
](C(=O)OC)[C@@]3(CC2)C)(C1)C
c1cocc1[C@H]1OC(=O)[C@H]2[C@@]([C@H]3C(=O)[C@@H](OC(=O)CC(=O)OCC)C[C@@
H](C(=O)OC)[C@@]3(CC2)C)(C1)C
c1ccc(c(c1)C(=O)O)C(=O)O[C@@H]1C(=O)[C@@H]2[C@@]3([C@H](C(=O)O[C@H](c4ccoc
4)C3)CC[C@]2([C@H](C(=O)OC)C1)C)C
c1cocc1[C@H]1OC(=O)[C@H]2[C@@]([C@H]3C(=O)[C@@H](OC(=O)CC(=O)OC(C)(C)C)C[
C@@H](C(=O)OC)[C@@]3(CC2)C)(C1)C
c1cocc1[C@H]1OC(=O)[C@H]2[C@@]([C@H]3C(=O)[C@@H](OC(=O)CCC(=O)OC)C[C@@
H](C(=O)OC)[C@@]3(CC2)C)(C1)C
c1cocc1[C@H]1OC(=O)[C@H]2[C@@]([C@H]3C(=O)[C@@H](OC(=O)CCCC(=O)OC)C[C@
@H](C(=O)OC)[C@@]3(CC2)C)(C1)C
c1cocc1[C@H]1OC(=O)[C@H]2[C@@]([C@H]3C(=O)[C@@H](OC(=O)CCC(=O)OCC)C[C@
@H](C(=O)OC)[C@@]3(CC2)C)(C1)C
c1cc(cc2cc(C(=O)N3CCN(CC3)S(=O)(=O)c3ccc(cc3[N+](=O)[O-])[N+](=O)[O-])[nH]c12)Cl
c1ccc(cc1)Nc1ccccc1C(=O)N1CCN(CC1)S(=O)(=O)c1c(c(cs1)C(=O)OC)OC
c1cocc1[C@H]1OC(=O)[C@H]2[C@@]([C@H]3C(=O)[C@@H](O)C[C@@H](C(=O)OC)[C@
@]3(CC2)C)(C1)C

c1ccc2c(c1)c1c([C@@H]3Oc4c5c(ccc4O)C[C@H]4N(CC[C@@]35[C@@]4(OCC)C1)CC=C)[nH]2
c1cc(c2c(c1N1CCN(C(=O)C[C@@H]3CNc4ccc(cc34)Br)CC1)non2)[N+](=O)[O-]
c1ccc(c(c1)C[C@H]1NCCC2=C1CC=C(C2)OC)O
c1cc(cc(c1c1cc([C@H]2CCN(C(=O)NCCn3c(cnc3C)[N+](=O)[O-])CC2)[nH]n1)Cl)Cl
C1=CC(=O)[C@@H]2[C@@]34[C@H]([C@H](N(C=O)CC3)C[C@H]2C1=O)CCC(=O)C4
c1ccc2c(c1)c1c([C@@H]3Oc4c5c(ccc4O)C[C@H]4N(CC[C@@]35[C@@]4(OCC)C1)CC1CC1)o2
c1cc2c3c(c1)O[C@H]1C(=O)CC[C@H]4[C@H](N(C=O)CC[C@]314)C2
c1cc(c2c(c1O)C[C@H]1N(C=O)CC[C@@]32[C@H]1CCC(=O)C3)O
c1ccc2c(c1)c1c([C@@H]3Oc4c5c(ccc4O)C[C@H]4N(CC[C@@]35[C@@]4(OC)C1)CC1CCCCC1)[nH]2
C(#N)c1cccc(c1)[C@]1(OC)[C@@H](CN(C)C)CCCC1
c1ccc2c(c1)c1c([C@@H]3Oc4c5c(ccc4O)C[C@H]4N(CC[C@@]35[C@@]4(OCC=C)C1)CC=C)n2CC=C
c1cc(cc(c1)OC)[C@@]1(O)[C@H](CN(C)CCNC(=O)c2cc(c(cc2OC)N)Cl)CCCC1
c1cc(cc(c1)OC)[C@]1(O)[C@@H](CN2CC[C@@H](NC(=O)c3cc(c(cc3OC)N)Cl)[C@@H](OC)C2)CCCC1
c1ccc2c(c1)c1c([C@@H]3Oc4c5c(ccc4O)C[C@H]4N(CC[C@@]35[C@@]4(OCc3ccccc3Cl)C1)CC=C)n2Cc1ccccc1Cl
c1cc(cc(c1)O)[C@]1(O)[C@@H](CN2CC[C@@H](NC(=O)c3cc(c(cc3OC)N)Cl)[C@@H](OC)C2)CCCC1
c1cc(cc(c1)OC)[C@]1(O)[C@@H](CN2CC[C@H](NC(=O)c3cc(c(cc3OC)N)Cl)[C@H](OC)C2)CCCC1
c1cc(cc(c1)O)[C@]1(O)[C@@H](CN2CC[C@H](NC(=O)c3cc(c(cc3OC)N)Cl)[C@H](OC)C2)CCCC1
c1cc(cc(c1)OC)[C@@]1(O)[C@H](CN2CC[C@H](NC(=O)c3cc(c(cc3OC)N)Cl)[C@H](OC)C2)CCCC1
c1cc(cc(c1)OC)[C@@]1(O)[C@H](CN2CC[C@@H](NC(=O)c3cc(c(cc3OC)N)Cl)[C@@H](OC)C2)CCCC1
c1cc(cc(c1)OC)[C@]1(OC)[C@@H](CNC)CCCC1
c1ccc(cc1)[C@]1(OC)[C@@H](CN(C)C)CCCC1
c1cc(cc(c1)OC)[C@@]1(OC)[C@H](CNC)CCCC1
c1ccc(cc1)[C@@]1(OC)[C@H](CN(C)C)CCCC1
c1cc(cc(c1)OC)[C@]1(OC)[C@@H](CN(C)C)CCCC1
c1cc(cc(c1)OC)[C@]1(OCC)[C@@H](CN(C)C)CCCC1
c1cc(cc(c1)O)[C@@]1(O)[C@H](CN2CC[C@@H](NC(=O)c3cc(c(cc3OC)N)Cl)[C@@H](OC)C2)CCCC1
c1cc(c2c3c1C[C@H]1N(CC[C@@]43[C@@]1(Br)C=CC(=O)[C@@H]4O2)C)OC
c1cc(cc(c1)OC)[C@@]1(O)[C@H](CN(C)C)CCCC1
C(#N)c1ccc(cc1)Oc1ccc(cc1)CCNCc1ccccc1
c1cc(c2c3c1C[C@H]1N(CC[C@]43[C@@H](O2)C2=C(C=C14)S(=O)(=O)CCN2)C)O
c1ccc(cc1)CNCCc1ccc(cc1)Oc1ccc(cc1)CN
c1cc(cc(c1)OC)[C@]1(O)[C@@H](CN(C)C)CCCC1
c1ccc(cc1)CNCCc1ccc(cc1)Oc1ccc(cc1)C(=O)O
c1ccc(cc1)CNCCc1ccc(cc1)Oc1ccc(cc1)S(=O)(=O)N
c1ccc(cc1)C(=C1CCN(CC1)C)c1ccc(cc1)C(=O)N(CC)CC

c1cc(c2c3c1C[C@H]1N(CC[C@]43[C@@H](O2)C(=C(C=C14)SSC1=C([C@H]2[C@]34C(=C1)[C@H](N(CC3)C)Cc1c4c(O2)c(cc1)O)NCC=O)NCC=O)C)O
c1ccc(cc1)C(=C1CCN(CC1)CCCC)c1ccc(cc1)C(=O)N(CC)CC
c1ccc(cc1)CN1CC[C@]23[C@]4(O)[C@H]1Cc1ccc(c(c21)O[C@H]3[C@@]1(O[C@H]2N3[C@H](OC2)CO[C@H]13)CC4)O
c1ccc(cc1)C(=C1CCN(CC1)CC1CC1)c1ccc(cc1)C(=O)N(CC)CC
c1ccc(cc1)/C(=C/1\CCN(c2ccccc2)CC1)/c1ccc(cc1)C(=O)N(CC)CC
c1cc(ccc1C(=C1CCNCC1)c1ccc(cc1)F)C(=O)N(CC)CC
c1cc(ccc1C(=C1CCNCC1)c1ccc(cc1)Cl)C(=O)N(CC)CC
c1cc(cc(c1)C(F)(F)F)C(=C1CCNCC1)c1ccc(cc1)C(=O)N(CC)CC
c1cc(ccc1C(=C1CCNCC1)c1ccc(c(c1)Cl)Cl)C(=O)N(CC)CC
c1cc(cc(c1)C(=O)C)C(=C1CCNCC1)c1ccc(cc1)C(=O)N(CC)CC
c1cc(cc(c1)[N+](=O)[O-])C(=C1CCNCC1)c1ccc(cc1)C(=O)N(CC)CC
c1cc(C(=C2CCNCC2)c2ccc(cc2)C(=O)N(CC)CC)sc1
c1ccc(c(c1)C(=C1CCNCC1)c1ccc(cc1)C(=O)N(CC)CC)F
c1cc(ccc1C(=C1CCNCC1)c1ccc(cc1)SC)C(=O)N(CC)CC
c1cc2c3c(c1C(=O)N(C)C)O[C@H]1[C@@H](O)C=C[C@H]4[C@H](N(CC[C@]314)C)C2
c1cc(cc(c1)N)C(=C1CCNCC1)c1ccc(cc1)C(=O)N(CC)CC
c1ccc(cc1)n1cc(c(C)n1)CN1CC[C@]2(c3ccccc3CO2)CC1
c1ccc(c(c1)C(=C1CCNCC1)c1ccc(cc1)C(=O)N(CC)CC)OC
c1cc(ccc1C(=C1CCNCC1)c1ccc(cc1)OC)C(=O)N(CC)CC
c1ccc2c(c1)CO[C@]12CCN(CC1)Cc1cn(c2c(cccn2)Cl)nc1C
c1ccc2c(c1)CO[C@]12CCN(CC1)Cc1cn(c2c(cccn2)C)nc1C
c1(ccc(cc1)[C@@H](N1C[C@H](N(C[C@@H]1C)CC=C)C)c1ccccc1)C(=O)N(CC)CC
c1cc(cc(c1)O)[C@]1([C@H](CN([C@H](CCN2CCCC2)C1)CCc1ccccc1)C)C
c1cc(ccc1)[C@]1([C@H](CN2[C@@H](CN([C@H](C2)c2ccccc2)Cc2ccccc2)C1)C)C
c1(ccc(cc1)N([C@H]1[C@H](CN(CC1)C/C=C/C)C)c1ccccc1)C(=O)N(CC)CC
c1(ccc(cc1)N([C@H]1[C@@H](CN(CC1)CC=C)C)c1ccccc1)C(=O)N(CC)CC
c1(ccc(cc1)N([C@@H]1[C@H](CN(CC1)CC=C)C)c1ccccc1)C(=O)N(CC)CC
c1(ccc(cc1)N([C@H]1[C@H](CN(CC1)CC=C)C)c1ccccc1)C(=O)N(CC)CC
c1(ccc(cc1)N([C@@H]1[C@@H](CN(CC1)CC=C)C)c1cc(ccc1)OC)C(=O)N(CC)CC
c1(=O)n(c2c(n1CC)cccc2)[C@@H]1[C@@H](CO)CN(CC1)CC1CCCCCCC1
c1ccc2c(c1)n(c(=O)n2CC)[C@@H]1[C@H](CO)CN(CC1)CC1CCCCCCC1
n1c(c(cc(c1)c1n(nc(c1C)CN[C@@H]1C[C@H](F)CC1)c1ncccc1Cl)F)C
c1(cc(cc(c1)c1n(nc(c1C)CN[C@@H]1C[C@H](F)CC1)CCC#N)F)F
c1ccc2c(c1)n(c(=O)n2C[C@@H](O)CO)[C@H]1CCN(CC1)C[C@@H]1C2([C@H]3C[C@@H]1CC3)CC2
c1ccc2c(c1)n(c(=O)n2C[C@H](O)CO)[C@H]1CCN(CC1)C[C@@H]1C2([C@H]3C[C@@H]1CC3)CC2
c1(ccc(cc1)[C@H](N1[C@@H]2[C@H]3N([C@@H]([C@H]1CC2)CC3)CC=C)c1cccc(c1)OC)C(=O)N(CC)CC
c1(ccc(cc1)[C@H](N1[C@@H]2[C@H]3N([C@@H]([C@H]1CC2)CC3)CC=C)c1cccc(c1)OC)C(=O)N(CC)C1CCCCC1
C1(=CCC2=C(C1)CCN[C@H]2Cc1c(cccc1)O)OC
c1ccc2c(c1)n(c(=O)n2C(CO)CO)[C@H]1CCN(CC1)C[C@@H]1C2([C@H]3C[C@@H]1CC3)CC2
c12ccc(cc1[C@]1([C@H]([C@H](N(CC3CC3)CC1)C2)C)C)C(=O)N(C)C

N1(CC[C@]23[C@]4(O)[C@H]1Cc1c2c(c(cc1)O)O[C@H]3[C@]1(O[C@@H]2N3[C@H]1OC([C@@H]3OC2(C)C)(C)C)CC4)Cc1ccccc1

Supplementary Section S5. Detailed description of the applied in silico tools.

Pharmacophore modeling deploys the spatial arrangement of electrochemical features to represent the binding mode of ligands for a specific target[46] and to identify novel molecules that display similar feature patterns. Shape-based screening, as the name already indicates, uses the 3D shape of a known active compound for model generation and filters compounds with similar shapes out of large compound databases. The shape-models can further be modified *via* addition of chemical features, which define again electrochemical properties.[47] 2D similarity-based methods compare the 2D structures of a set of known active compounds to a query molecule and employ statistical methods, based on the structural similarity, to calculate the probability that the compound of interest may also interact with the target. Docking is the only method that explicitly requires structural information on the target, e.g. derived from X-ray crystallography, NMR, or homology/molecular dynamics modeling. Basically, it comprises of two steps: first, the compounds of interest are fitted into the empty binding pocket of the target, and in a second step, the goodness of the binding pose in terms of binding energy is estimated *via* calculation of a fitness score.[21]

Pharmacophore modeling

All pharmacophore modeling studies were performed with LigandScout version 3.1[25]. This program allows for both structure- and ligand-based modeling. In case a structure-based approach is employed, the program automatically calculates the interaction pattern present in the crystal complex. This initial model can then be refined with the “actives” and “inactives” dataset. In the course of ligand-based modeling, conformations of two or more known active are aligned, and common pharmacophore features are extracted.[25] Again, this initial model can then be manually refined. As default, LigandScout uses HBD, HBA, metal binding (M), PI, negatively (NI) ionizable, Aro, and H features. In addition, steric

constraints (XVols) can be added to a model to prevent mapping of compounds that would probably clash with the binding site.[25]

All datasets and the Maybridge database were converted to 3D multiconformational databases using the Generate Library protocol (OMEGA version 2.3.3[48-50]) implemented in LigandScout. A maximum number of 50 conformers were generated for every molecule. Only compounds mapping all features included in the model were considered as hits.

After the prospective virtual screening, an additional molecular weight filter was applied. The molecular weight of the agonists or antagonists was calculated with Discovery Studio, and the average ± the standard deviation was used as filter. For agonists, the molecular weight ranged from 316 - 473, whereas the antagonist hitlist was filtered for compounds with a molecular weight from 344 - 506.

Finally, all hits were ranked according to the relative pharmacophore fit value, and the top 4-ranked agonists, and the top-2 ranked antagonists were selected for biological testing.

Shape-based modeling

The software program vROCS version 3.0.0[23,24] was employed for shape-based modeling. This program applies atom-centered Gaussian functions to calculate the shape-overlap of a query molecule and the model. In addition, so-called color features can be added. As default, HBD, HBA, R, H, anionic (A), and C colour features are available. Automatically generated models derived from the co-crystallized ligand or one low-energy conformation of the query molecules calculated with OMEGA 2.3.2[49-51] were further optimized with the "actives" and "inactives" dataset. The default Implicit-Mills-Dean force-field was applied for virtual screening, and the ComboScore was selected as scoring function.

The hit lists were filtered with the same molecular weight filter applied in the pharmacophore-based studies. In the course of a ROCS-based virtual screening run, a score is assigned for all compounds up to a user-defined number, no matter if the database compounds match all features or not. To discriminate

between active and inactive compounds, an activity cut-off was defined for every model during the theoretical model validation. These values can be quite distinct, as the probability that compounds match the majority of the features largely depends on the number and composition of the features. A model with a low number of features therefore more often retrieves compounds with a very high ComboScore compared to a complex model with multiple color features. To account for this bias, we normalized the retrieved ComboScore with the validation cut-off, and used this normalized ComboScore for ranking of the compounds in the prospective screening. Finally, the four top-ranked compounds from the agonist hitlist and the top-2 ranked compounds from the antagonist hitlist were selected for biological testing.

All datasets and the Maybridge database were converted to an oeb.gz file using OMEGA 2.3.2 and the default settings, leading to a maximum number of 200 conformers per molecules.

Docking

All docking studies were performed with GOLD version 5.2.[52] This program uses a genetic algorithm to calculate up to ten docking poses per input-ligand. The GoldScore was selected as fitness score, which takes hydrogen bonding, ligand internal strains, and steric aspects of the receptor-ligand complex into account.[53]

The crystal structure of the β-FNA -MOR complex (PDB-entry 4DKL[1]) was used for the docking studies. In the course of the protein preparation, hydrogens were added, and all water molecules except for 718 and 719 were deleted. The remaining two water molecules were set to "toggle and spin". This allowed the program to automatically decide whether or not a water molecule is included during docking, and to optimize the orientation of the water molecule. The area of 6 Å around the co-crystallized ligand was defined as binding site, and a protein hydrogen bond with the phenolic hydroxyl-group of Tyr148 was defined as constraint. Using these parameters, β-FNA was re-docked into the empty binding pocket of the MOR with an RMSD-value of 1.4330 Å. This RMSD-value might appear high at first sight,

however, a covalently attached ligand was docked using a "non-covalent" docking protocol. We decided for a non-covalent docking procedure, because the majority of known ligands and all of the compounds in our datasets were competitive binders. The double bond involved in the chemical reaction is about 3.6 Å apart from Lys233 in the docking pose, suggesting an appropriate position for the reaction. One low-energy conformation per molecule calculated with OMEGA 2.3.2[49-51] served as input file for docking.

Since antagonists tended to have a higher GoldScore, two different cut-off values were defined for agonists and antagonists. Every agonist with a GoldScore ≥ 44.0 and antagonists with a GoldScore of ≥ 48.0 were considered as active. The interaction patterns of the highest ranked docking pose served as discrimination between agonists and antagonists.

In the prospective screening, a maximum number of ten docking poses for each of the ~ 52,000 compounds in the Maybridge database was derived. To limit the amount of data, the results were filtered according to the GoldScore, where all results above a GoldScore of 65.0 were kept. In addition this file was filtered one time with the agonist-molecular weight filter for potential novel agonist, and one time with the antagonist-molecular weight filter to identify novel antagonists, respectively. Both hitlists were then ranked according to the GoldScore, and the interaction patterns of the docked compounds were visually inspected. The four and two compounds with the highest GoldScore that fulfilled the required interaction patterns were selected for the biological evaluation as MOR agonists and antagonists, respectively.

Bioactivity profiling tools

2D-smilarity-based profiling

SEA. SEA compares the 2D structure of a query molecule with the 2D structures of a set of already known active compounds to calculate the probability that the molecule of interest also interacts with the same target. The program employs the ECFP4 as descriptor for the 2D structures of molecules, and the

Tc[54,55] to determine the similarity between pairs of compounds. To avoid "random similarity" of compounds, SEA applies a statistical model that calculates expectation values (E-values). The lower the E-value, the higher is the probability for an interaction. Several databases are available as "actives" dataset for comparison. We used the ChEMBL version 16, the MDDR classes that have known targets database (version 2006.1), the WOMBAT database (version 2006.2), and the KEGG version 2008 dataset. The sd-file of all selected virtual hits was converted to the smiles code with OpenBabel,[56,57] and each single compound was subjected to the profiling. Analogous to our previous studies, we also applied an activity cut-off of an E-value ≤ -4 within this study, as proposed by Lounkine et al.[58]

PASS. PASS relies on the same principle as SEA, but utilizes the Multilevel Neighborhoods of Atoms (MNA) descriptor, and an improved naïve Bayes algorithm for calculating the probabilities of activity and inactivity. The active dataset contains only known active compounds that have been manually collected from original literature.[18] Similar to SEA, the smiles code of every compound after the other was uploaded for the prediction. Analogous to our previous studies,[22,28] the Probability to be active (Pa) of ≥ 0.5 was defined as activity cut-off. Poroikov et al. showed that the majority of active compounds is distributed above that cut-off.[59]

Pharmacophore-based profiling

PharmMapper. The PharmMapper model collection contains more than 7,000 structure-based pharmacophore models covering approximately 1,600 drug targets. Although the models have been automatically generated with LigandScout, an in-house virtual screening algorithm was employed.[19] One single sd-file was uploaded for every compound, and a maximum number of 300 conformers was generated. The number of results was limited to the 300 best-matching pharmacophore models. To ensure a sufficient restrictivity, only models with ≥ 6 pharmacophore features were considered as hits.

PharmaDB. In our previous studies,[22,28] we also included the commercial pharmacophore model collection PharmaDB, which was implemented in Discovery Studio. The models were automatically generated from target-ligand complexes deposited in the Protein Data Bank, and the restrictivity of the created models was estimated with a trained genetic function approximation model. A number of 3 to 6 pharmacophore features was allowed for every model, and a maximum number or ten models per complex was selected for the collection.[20] Unfortunately, this model collection does not contain any MOR models, however, for the sake of completeness we considered it as an added-value to the present study.

Hardware specification. All processes and predictions were performed on a multi-core workstation with 2.4+ GHz, 8 GB of RAM, a 1+TB fast mass storage, and a NVIDIA graphical processing unit. All programs run on the Windows 7 platform.

REFERENCES

1 Manglik, A. *et al.* Crystal structure of the μ-opioid receptor bound to a morphinan antagonist. *Nature* **485**, 321-326 (2012).

2 Spetea, M. & Schmidhammer, H. Recent advances in the development of 14-alkoxy substituted morphinans as potent and safer opioid analgesics. *Curr. Med. Chem.* **19**, 2442-2457 (2012).

3 Ananthan, S. *et al.* 14-Alkoxy- and 14-acyloxypyridomorphinans: μ Agonist/δ antagonist opioid analgesics with diminished tolerance and dependence side effects. *J. Med. Chem.* **55**, 8350-8363 (2012).

4 Wentland, M. P. *et al.* Syntheses and opioid receptor binding properties of carboxamido-substituted opioids. *Bioorg. Med. Chem. Lett.* **19**, 203-208 (2009).

5 Schmidhammer, H. & Spetea, M. in *Chemistry of Opioids* Vol. 299 *Topics in Current Chemistry* (ed Hiroshi Nagase) Ch. 77, 63-91 (Springer Berlin Heidelberg, 2011).

6 Schüllner, F. *et al.* Synthesis and biological evaluation of 14-alkoxymorphinans. Part 19. *Helv. Chim. Acta* **86**, 2335-2341 (2003).

7 Le Bourdonnec, B. *et al.* Trans-3,4-dimethyl-4-(3-carboxamidophenyl)piperidines: A novel class of μ-selective opioid antagonists. *Bioorg. Med. Chem. Lett.* **13**, 4459-4462 (2003).

8 Le Bourdonnec, B. *et al.* Novel trans-3,4-dimethyl-4-(3-hydroxyphenyl)piperidines as μ opioid receptor antagonists with improved opioid receptor selectivity profiles. *Bioorg. Med. Chem. Lett.* **18**, 2006-2012 (2008).

9 Le Bourdonnec, B. *et al.* Elucidation of the bioactive conformation of the N-substituted trans-3,4-dimethyl-4-(3-hydroxyphenyl)piperidine class of μ-opioid receptor antagonists. *J. Med. Chem.* **49**, 7278-7289 (2006).

10 Le Bourdonnec, B. *et al.* Synthesis and pharmacological evaluation of novel octahydro-1H-pyrido[1,2-a]pyrazine as μ-opioid receptor antagonists. *J. Med. Chem.* **49**, 7290-7306 (2006).

11 Carroll, F. I. *et al.* 4β-Methyl-5-(3-hydroxyphenyl)morphan opioid agonist and partial agonist derived from a 4β-methyl-5-(3-hydroxyphenyl)morphan pure antagonist. *J. Med. Chem.* **56**, 8826-8833 (2013).

12 Schmidt, H. *et al.* Affinities of dihydrocodeine and its metabolites to opioid receptors. *Pharmacol. Toxicol.* **91**, 57-63 (2002).

13 Neumeyer, J. L. *et al.* Synthesis, binding affinity, and functional in vitro activity of 3-benzylaminomorphinan and 3-benzylaminomorphine ligands at opioid receptors. *J. Med. Chem.* **55**, 3878-3890 (2012).

14 Takeuchi, K. *et al.* Structure–activity relationship studies of carboxamido-biaryl ethers as opioid receptor antagonists (OpRAs). Part 1. *Bioorg. Med. Chem. Lett.* **17**, 5349-5352, (2007).

15 Wentland, M. P. *et al.* Syntheses of novel high affinity ligands for opioid receptors. *Bioorg. Med. Chem. Lett.* **19**, 2289-2294 (2009).

16 Rennison, D., Moynihan, H., Traynor, J. R., Lewis, J. W. & Husbands, S. M. Structural determinants of opioid activity in derivatives of 14-aminomorphinones: Effects of changes to the chain linking of the C14-amino group to the aryl ring. *J. Med. Chem.* **49**, 6104-6110 (2006).

17 Keiser, M. J. *et al.* Relating protein pharmacology by ligand chemistry. *Nat. Biotechnol.* **25**, 197-206 (2007) (Access dates 20 August 2014, 24, 27, and 30 October 2014, and 12-13 November 2104).

18 Filimonov, D. A. *et al.* Prediction of the biological activity spectra of organic compounds using the Pass online web resource. *Chem. Heterocycl. Compd.* **50**, 444-457 (2014) (Access dates 14, 18, and 20 August 2014).

19 Liu, X. *et al.* PharmMapper server: a web server for potential drug target identification using pharmacophore mapping approach. *Nucleic Acids Res.* **38**, W609-614 (2010) (Access dates 20 and 22 August 2014).
20 Meslamani, J. *et al.* Protein-ligand-based pharmacophores: generation and utility assessment in computational ligand profiling. *J. Chem. Inf. Model.*, 943-955 (2012).
21 Kitchen, D. B., Decornez, H., Furr, J. R. & Bajorath, J. Docking and scoring in virtual screening for drug discovery: methods and applications. *Nat. Rev. Drug Discovery* **3**, 935-949 (2004).
22 Kaserer, T. *et al.* Prospective performance evaluation of selected common virtual screening tools. Case study: cyclooxygenase (COX) 1 and 2. *Eur. J. Med. Chem.* **96**, 445-457 (2015).
23 vROCS version 3.0.0, OpenEye Scientific Software, Santa FE, NM, http://www.eyesopen.com.
24 Hawkins, P. C., Skillman, A. G. & Nicholls, A. Comparison of shape-matching and docking as virtual screening tools. *J. Med. Chem.* **50**, 74-82 (2007).
25 Wolber, G. & Langer, T. LigandScout: 3-D pharmacophores derived from protein-bound ligands and their use as virtual screening filters. *J. Chem. Inf. Model.* **45**, 160-169 (2005).
26 Krüger, D. M. & Evers, A. Comparison of structure- and ligand-based virtual screening protocols considering hit list complementarity and enrichment factors. *ChemMedChem* **5**, 148-158 (2010).
27 Gaulton, A. *et al.* ChEMBL: a large-scale bioactivity database for drug discovery. *Nucleic Acids Res.* **40**, D1100-D1107 (2012).
28 Kaserer, T. *et al.* In silico predictions of drug-drug interactions caused by CYP1A2, 2C9, and 3A4 inhibition - a comparative study of virtual screening performance. *Mol. Inf.* **34**, 431– 457 (2015).
29 Huang, W. *et al.* Structural insights into µ-opioid receptor activation. *Nature* **524** 315-321, (2015).
30 Wu, H. *et al.* Structure of the human κ-opioid receptor in complex with JDTic. *Nature* **485**, 327-332 (2012).
31 Schmidhammer, H. *et al.* Functionalization of the carbonyl group in position 6 of morphinan-6-ones. Development of novel 6-amino and 6-guanidino substituted 14-alkoxymorphinans. *Curr. Pharm. Des.* **19**, 7391-7399 (2013).
32 Fujii, H. Twin and triplet drugs in opioid research. *Top. Curr. Chem.* **299**, 239-275, (2011).
33 Fujimoto, R. A. *et al.* Synthesis, opioid receptor binding profile, and antinociceptive activity of 1-azaspiro[4.5]decan-10-yl amides. *J. Med. Chem.* **32**, 1259-1265 (1989).
34 Greedy, B. M. *et al.* Orvinols with Mixed Kappa/Mu Opioid Receptor Agonist Activity. *J. Med. Chem.* **56**, 3207-3216 (2013).
35 Greiner, E. *et al.* Synthesis and biological evaluation of 14-alkoxymorphinans. 18.1 N-Substituted 14-phenylpropyloxymorphinan-6-ones with unanticipated agonist properties: Extending the scope of common structure–activity relationships. *J. Med. Chem.* **46**, 1758-1763 (2003).
36 Lovell, K., Prevatt-Smith, K., Lozama, A. & Prisinzano, T. in *Chemistry of Opioids* Vol. 299 *Topics in Current Chemistry* (ed Hiroshi Nagase) Ch. 82, 141-185 (Springer Berlin Heidelberg, 2011).
37 Peng, X., Knapp, B. I., M.Bidlack, J. & Neumeyer, J. L. High Affinity of Carbamate Analogues of Morphinan atOpioid Receptors. *Bioorg. Med. Chem. Lett.* **17**, 1508-1511 (2007).
38 Mosberg, H. I. *et al.* Opioid peptidomimetics: Leads for the design of bioavailable mixed efficacy µ opioid receptor (MOR) agonist/δ opioid receptor (DOR) antagonist ligands. *J. Med. Chem.* **56**, 2139-2149 (2013).
39 Shao, L. *et al.* In vitro and in vivo evaluation of O-alkyl derivatives of tramadol. *Bioorg. Med. Chem. Lett.* **18**, 1674-1680 (2008).

40 Sipos, A. *et al.* Synthesis and characterization of thiazolo- and thiazinomorphinans and their intermediate products as novel opioid-active derivatives. *Arch. Pharm. Chem. Life Sci.* **345**, 852-858 (2012).
41 Wada, N. *et al.* Synthesis of novel triplet drugs with 1,3,5-trioxazatriquinane skeletons and their pharmacologies. 3: Synthesis of novel triplet drugs with the bis(epoxymethano) or bis(dimethylepoxymethano) structure (double-capped triplet). *Bioorg. Med. Chem. Lett.* **22**, 7551-7554 (2012).
42 Iyer, M. R., Rothman, R. B., Dersch, C. M., Jacobson, A. E. & Rice, K. C. Probes for narcotic receptor mediated phenomena. 47.(1) Novel C4a- and N-substituted-1,2,3,4,4a,9a-hexahydrobenzofuro[2,3-c] pyridin-6-ols. *Bioorg. Med. Chem.* **21**, 3298-3309, (2013).
43 Lunn, G. *et al.* SAR and biological evaluation of 3-azabicyclo[3.1.0]hexane derivatives as μ opioid ligands. *Bioorg. Med. Chem. Lett.* **22**, 2200-2203 (2012).
44 VanAlstine, M. A. *et al.* Redefining the structure–activity relationships of 2,6-methano-3-benzazocines. Part 9: Synthesis, characterization and molecular modeling of pyridinyl isosteres of N-BPE-8-CAC (1), a high affinity ligand for opioid receptors. *Bioorg. Med. Chem. Lett.* **23**, 2128-2133 (2013).
45 Wentland, M. P. *et al.* Redefining the structure–activity relationships of 2,6-methano-3-benzazocines. Part 8. High affinity ligands for opioid receptors in the picomolar Ki range: Oxygenated N-(2-[1,1′-biphenyl]-4-ylethyl) analogues of 8-CAC. *Bioorg. Med. Chem. Lett.* **22**, 7340-7344 (2012).
46 Wermuth, G., Ganellin, C. R., Lindberg, P. & Mitscher, L. A. Glossary of terms used in medicinal chemistry (IUPAC Recommendations 1998). *Pure Appl. Chem.* **70**, 1129-1143 (1998).
47 Kirchmair, J. *et al.* How to optimize shape-based virtual screening: choosing the right query and including chemical information. *J. Chem. Inf. Model.* **49**, 678-692 (2009).
48 OMEGA version 2.3.3, OpenEye Scientific Software, Santa Fe, NM, http://www.eyesopen.com.
49 Hawkins, P. C., Skillman, A. G., Warren, G. L., Ellingson, B. A. & Stahl, M. T. Conformer generation with OMEGA: algorithm and validation using high quality structures from the Protein Databank and Cambridge Structural Database. *J. Chem. Inf. Model.* **50**, 572-584 (2010).
50 Hawkins, P. C. & Nicholls, A. Conformer generation with OMEGA: learning from the data set and the analysis of failures. *J. Chem. Inf. Model.* **52**, 2919-2936 (2012).
51 OMEGA version 2.3.2, OpenEye Scientific Software, Sante FE, NM, http://www.eyesopen.com.
52 GOLD version 5.2, CCDC, Cambridge, UK, www.ccdc.cam.ac.uk.
53 Jones, G., Willett, P., Glen, R. C., Leach, A. R. & Taylor, R. Development and validation of a genetic algorithm for flexible docking. *J. Mol. Biol.* **267**, 727-748 (1997).
54 Jaccard, P. Distribution de la flore alpine dans le bassin des Dranses et dans quelques régions voisines. *Bull. Soc. Vaud. sci. nat.* **37**, 241-272 (1901).
55 Rogers, D. J. and Tanimoto, T. T. A computer program for classifying plants. Science 132, 1115-1118 (1960)
56 The Open Babel Package v. 2.3.1. http://openbabel.org.
57 O'Boyle, N. *et al.* Open Babel: An open chemical toolbox. *J. Cheminf.* **3**, 33 (2011).
58 Lounkine, E. *et al.* Large-scale prediction and testing of drug activity on side-effect targets. *Nature* **486**, 361-367 (2012).
59 Poroikov, V., Akimov, D., Shabelnikova, E. & Filimonov, D. Top 200 medicines: can new actions be discovered through computer-aided prediction? *SAR QSAR Environ. Res.* **12**, 327-344 (2001).
